# Efficacy of a Multi-level Intervention to Reduce Injecting and Sexual Risk Behaviors among HIV-Infected People Who Inject Drugs in Vietnam: A Four-Arm Randomized Controlled Trial

**DOI:** 10.1371/journal.pone.0125909

**Published:** 2015-05-26

**Authors:** Vivian F. Go, Constantine Frangakis, Nguyen Le Minh, Carl Latkin, Tran Viet Ha, Tran Thi Mo, Teerada Sripaipan, Wendy W. Davis, Carla Zelaya, Pham The Vu, David D. Celentano, Vu Minh Quan

**Affiliations:** 1 Department of Health Behavior, University of North Carolina Gillings School of Global Public Health, Chapel Hill, North Carolina, United States of America; 2 Department of Biostatistics, Johns Hopkins Bloomberg School of Public Health, Baltimore, Maryland, United States of America; 3 Thai Nguyen Center for Preventive Medicine, Thai Nguyen, Vietnam; 4 Department of Health, Behavior and Society, Johns Hopkins Bloomberg School of Public Health, Baltimore, Maryland, United States of America; 5 Department of Epidemiology, Johns Hopkins Bloomberg School of Public Health, Baltimore, Maryland, United States of America; University of Washington, UNITED STATES

## Abstract

**Introduction:**

Injecting drug use is a primary driver of HIV epidemics in many countries. People who inject drugs (PWID) and are HIV infected are often doubly stigmatized and many encounter difficulties reducing risk behaviors. Prevention interventions for HIV-infected PWID that provide enhanced support at the individual, family, and community level to facilitate risk-reduction are needed.

**Methods:**

455 HIV-infected PWID and 355 of their HIV negative injecting network members living in 32 sub-districts in Thai Nguyen Province were enrolled. We conducted a two-stage randomization: First, sub-districts were randomized to either a community video screening and house-to-house visits or standard of care educational pamphlets. Second, within each sub-district, participants were randomized to receive either enhanced individual level post-test counseling and group support sessions or standard of care HIV testing and counseling. This resulted in four arms: 1) standard of care; 2) community level intervention; 3) individual level intervention; and 4) community plus individual intervention. Follow-up was conducted at 6, 12, 18, and 24 months. Primary outcomes were self-reported HIV injecting and sexual risk behaviors. Secondary outcomes included HIV incidence among HIV negative network members.

**Results:**

Fewer participants reported sharing injecting equipment and unprotected sex from baseline to 24 months in all arms (77% to 4% and 24% to 5% respectively). There were no significant differences at the 24-month visit among the 4 arms (Wald = 3.40 (3 df); p = 0.33; Wald = 6.73 (3 df); p = 0.08). There were a total of 4 HIV seroconversions over 24 months with no significant difference between intervention and control arms.

**Discussion:**

Understanding the mechanisms through which all arms, particularly the control arm, demonstrated both low risk behaviors and low HIV incidence has important implications for policy and prevention programming.

**Trial Registration:**

ClinicalTrials.gov NCT01689545

## Introduction

As of 2013, an estimated 1.6 million people who inject drugs (PWID) were infected with HIV [1] representing a global HIV prevalence of 11.5% among PWID. Outside of sub-Saharan Africa, about a third of HIV infections can be attributed to injection drug use while in Africa, drug use is becoming an increasingly important driver of the epidemic with more than a third of PWID HIV infected in some African countries [2]. Injecting and sexual risk behaviors among HIV-infected PWID [3, 4] place injecting and sexual partners at considerable risk for HIV [5, 6]. While many PWID decrease their risk behaviors after learning that they are HIV-infected [7–9], a significant proportion continue to engage in, or relapse to, unsafe sexual and injection risk behaviors [10–12]. Prevention interventions for HIV-infected PWID that provide enhanced support at the individual, family and community level to facilitate risk-reduction are needed.

Like many countries in Asia and Eastern Europe, the HIV epidemic in Vietnam is concentrated primarily among PWID. Estimates of HIV prevalence among PWID in Vietnam vary. According to the 2009 Integrated Biological and Behavioral Surveillance (IBBS), of the approximately 271,000 PWID in Vietnam, up to 40% were estimated to be HIV-infected [13]. HIV sentinel surveillance suggests that HIV prevalence among PWID decreased from 28.6% in 2004 to 10.6% in 2013, however numbers vary widely by province with 34% of PWID in the northern province of Thai Nguyen reported to be living with HIV in 2013 [14].

As in many other settings where injection drug use is the primary driver of a concentrated HIV epidemic, PWID face severe social marginalization within families and communities [15, 16], may be subject to compulsory detoxification and incarceration, and are discriminated against in health care settings [17]. As a result, they are difficult to reach for intervention and care and treatment programs [18]. In addition, HIV stigma in Vietnam may discourage people living with HIV/AIDS (PLWHA) from disclosing their HIV status to others, which can be a profound barrier to engaging in HIV prevention, care and support activities [19]. Despite recent efforts on behalf of the Vietnamese government to tackle HIV-related stigma through legislation [20], studies among the general population have found that most people remain afraid of and uncertain about HIV transmission. In our previous research in Vietnam, HIV infected PWID have indicated that stigma and social isolation are major barriers to HIV prevention efforts [21].

In concentrated epidemics, such as Vietnam’s, transmission may be seen as a “product of the social situations and environments in which individuals participate” [22]. A risk environment framework recognizes that risk behaviors are shaped by social, physical and political environments [22–24] and that effective HIV risk reduction interventions need to address multiple levels [25]. A review of individual, structural, and combination interventions for PWID found that while individual and structural interventions alone can achieve modest reductions in HIV transmission among PWID, combined approaches are likely to be most effective at preventing HIV transmission [26]. Similarly, Strathdee et al [27] showed through modeling that in settings where HIV epidemics are concentrated among PWID, the benefits of combination interventions were amplified by structural interventions that optimized efficacy and access to services. Despite evidence underscoring the need for multi-level interventions, to our knowledge, there has not been a multi-level study focusing on a key population such as PWID in a concentrated epidemic.

In settings such as Vietnam’s, stigma and lack of social support can pose considerable barriers to sexual and injecting risk reduction among HIV-infected PWID. Individuals may not get tested, may not seek care and/or may not disclose to risk partners, all of which have been associated with risk reduction [28, 29]. We used a risk environment approach to develop and evaluate a behavioral intervention for HIV-infected PWID. Specifically, we hypothesized that a multi-level intervention combining an individual level component that provides support, risk-reduction skills, and resilience to stigma with a structural-level component that aimed to reduce HIV and injecting drug use (IDU)-related stigma in the community would reduce HIV injecting and sexual risk behaviors compared to each component alone or the standard of care.

## Materials and Methods

The protocol for this trial and supporting CONSORT checklist are available as supporting information; see S1 CONSORT Checklist and S1 Protocol.

### Ethics statement

The study was approved by the ethical review committees at the Thai Nguyen Center for Preventive Medicine on April 23, 2009 and at the Johns Hopkins Bloomberg School of Public Health on June 10, 2009. Since the goal of the study was to assess the efficacy of a behavioral intervention and not to test drugs, biologics or devices, it was not registered in the ClinicalTrials.gov registry at the time of recruitment. However, once we were made aware that that our study was eligible for registration in 2012, it was registered at ClinicalTrials.gov (NCT01689545). The authors confirm that all ongoing and related trials for this intervention are registered.

### Study site

The study was conducted in Thai Nguyen, a northeastern province close to the border of China with a tradition of opium cultivation and use. Gold and tin mines attract migrant workers and the combination of urbanization and easily accessible opium and heroin has contributed to a rapid increase in injecting drug use since the mid-1990s. In 2014, there were an estimated 5848 PWID in the province [30]. The HIV prevalence among PWID was 33% in 2005 [31]. New needles and syringes are cheap and widely available in pharmacies in Thai Nguyen and it is legal for pharmacies to sell them to drug users. At the time of the study there were 16 needle exchange sites throughout Thai Nguyen.

### Design, participants, randomization and assessment procedures

We evaluated our multi-level intervention using a 2x2 (four-arm) factorial randomized controlled trial design consisting of: 1) standard of care HIV testing and counseling (HTC); 2) structural-level community stigma reduction program; 3) individual-level posttest counseling and skill-building support groups; 4) both individual and structural level activities. Our primary hypothesis was that combined individual and structural level activities are more effective in reducing injecting and sexual HIV risk behaviors than either an individual level activity or a structural level activity alone. HIV incidence among HIV–negative network members was a secondary outcome. Sample size calculations were conducted to determine the minimum number of HIV-infected PWID that would need to detect a 40% or 50% decrease in sexual and injection risks, accounting for a 20% drop-out rate. Power calculations assumed intention-to-treat distributions, 85% power and an alpha of. 05. A sample size of 404 will enable us to detect a decrease in frequency of unprotected sex of. 40 or above (assuming the stigma reduction programs were as effective as the individual-level intervention;. 50 or above if it was 50% as effective) for analyses on PWID. If the variance covariance parameters are similar between the sexual and drug risk trajectories, we can also detect a decrease in direct and indirect sharing of. 40 or above for analyses on PWID.

For our secondary hypothesis, that network members of indexes assigned the individual and structural level interventions will have decreased HIV incidence, we estimated that a sample size of 400 (200 combined sample size for the arm receiving both interventions and the arm receiving both controls) HIV-negative network members would enable us to assess a decrease in 24-month HIV incidence of between 20–25% or more accounting for a 20% drop-out rate (160 x 2/ (1-20/100)), with 80% power and alpha of. 05 for analyses on injecting network members.

**Fig 1:**
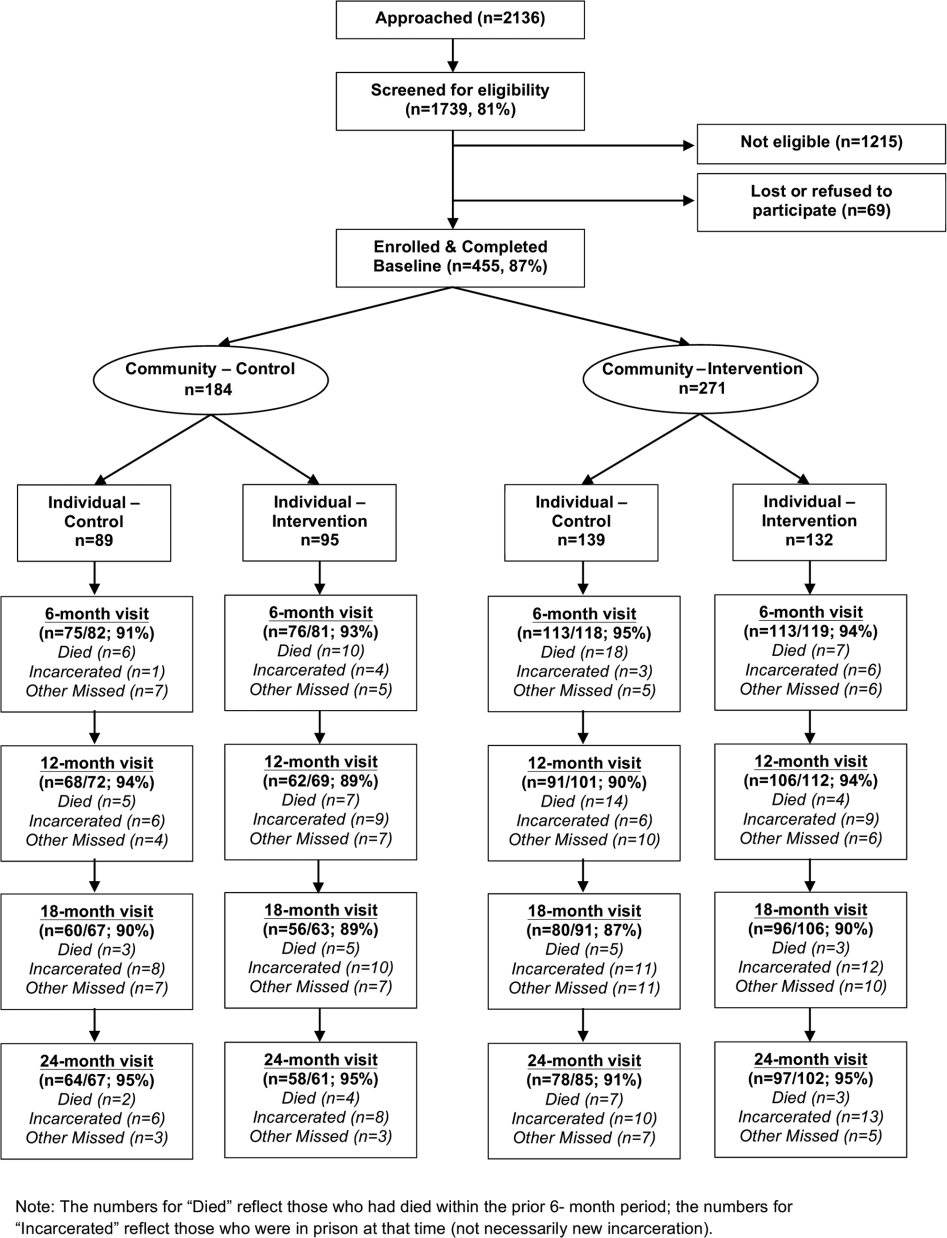
Consort index participant flow diagram (n = 455). Chronological schematic diagram showing number approached, screened, eligible, enrolled, randomized and lost to follow-up by arm at 6, 12, 18, and 24-month visits. Retention rates were calculated excluding those who died or were incarcerated from the denominator.

The four arms were constructed as follows. Out of a potential 180 sub-districts, where the average population in each is approximately 10,000, we selected the 32 sub-districts in Thai Nguyen that had the largest number of PWID. We then matched sub-districts that had about the same number of PWID and that were geographically distributed to minimize contamination between arms. Then, by the toss of a coin, we randomly selected 1 of each pair of sub-districts to receive the structural-level stigma reduction programs and the remaining 16 sub-districts to receive the standard of care. Within each of the 32 sub-districts (regardless of structural-level stigma reduction assignment) a random half of the PWID indexes were assigned to receive the enhanced posttest counseling and skill building support group (individual level intervention) and the other half the standard of care (see Fig 1). Because the number of HIV-infected PWID within each sub-district varied, the sample size of each arm was different. A computer program was used to assign conditions based on block randomization (n = 12). Sealed envelopes containing pre-computed blocks with 1:1 randomization to control and intervention were generated by computer and used in sequence for each group randomization. The arm was randomly assigned to index participants by matching ordered study ID numbers assigned at time of screening to the list of pre-computed assignments.

Index participants were recruited through a team of recruiters (n = 7) consisting of former and current drug users. Using a snowball sampling technique, recruiters approached their current or former drug networks in a private place, distributed brochures, and answered questions about our study. They then accompanied or referred interested subjects to the study site for screening. Inclusion criteria were: 1) an HIV-infected diagnosis confirmed through testing in our study; 2) able and willing to bring in an injecting network member for screening; 3) male (note: 97% of PWID in Thai Nguyen are male and female PWID typically have different risk factors.); 4) at least 18 years old; 5) had sex in the past 6 months; 6) injected drugs in the past 6 months; and 7) planned to live in Thai Nguyen for the next 2 years. Due to the challenge in identifying individuals who met these criteria, in order to reach our target sample size, we deviated from our original protocol in two ways. First, we increased the number of sub-districts from 8 to 32. Second, after approximately the first year of recruitment, we lifted the eligibility criteria for indexes to have a network member enrolled in the study. Eligibility criteria for network members were: 1) at least 18 years old; 2) HIV-negative; 3) injected drugs with the index participant in the past 6 months; and 4) interacted at least once a week with the index. Recruitment and follow-ups for the trial occurred between July 2009 and April 2013.

At screening, participants were tested for HIV antibody, counseled, and administered a face-to-face interview using a structured questionnaire. We used 2 rapid EIA tests run simultaneously (Determine: Abbot Laboratories, Abbott Park, IL and Bioline: SD, Toronto Canada) [32]. Discordant results were resolved through a third rapid assay (HIV Rapid Test: ACON, San Diego, CA). Although we provided results at the screening visits, in order to give individuals the opportunity to process the results of their tests, we enrolled participants at a later visit. A short pre-intervention survey was administered to all participants at enrollment to assess disclosure and dimensions of stigma after being diagnosed with HIV. Written informed consent was obtained from eligible participants at both the screening and enrollment visits.

Follow-up interviews were conducted among all index PWID participants at 6, 12, 18 and 24 months, and among network PWID participants at 12 and 24 months. The questionnaire was administered through face-to-face interviews in a private room at the project facility with trained interviewers and took approximately 1–1 ½ hours to complete. At baseline and each follow-up visit, index participants were asked to provide blood specimens in order to assess CD4 cell count. In addition, HIV testing and counseling was conducted among network participants at baseline and at the 12 and 24-month visits. Participants were reimbursed 75,000 Vietnamese Dong (VND), which is equivalent to $3.50 USD, at each visit plus 5000 VND ($0.23) for each kilometer traveled.

### Interventions

The multi-level intervention draws on theories of social action, social identity [33], and diffusions of innovation [34]. Social action theory, which centers on the interaction between internal affective states (e.g., internalized stigma and avoidance coping), the social environmental context (e.g., peer networks, sexual partners, community) and self-regulation capabilities (e.g., mastery of technical, social and problem solving skills), guided intervention content. Further, in accordance with the tenets of social identity theory we included small group sessions for individuals receiving our individual intervention. We hypothesized that a link between the self and the group would emerge and that an individual participant’s behavior would become aligned with group goals and actions to “be safe.” Finally, our structural level intervention used a community mobilization approach based on the diffusion of innovation theory [34]. In this community-based theory, a small number of people serve as innovators who influence behavioral change in others in a social network. Eventually, a threshold of behavioral adoption at the network level is reached which sustains the widespread uptake of an attitude or behavior. The combination of these three theories guided the content and delivery of our multi-level intervention.

#### Individual level intervention

Participants randomized to the individual level control arm received a pre-and post-test standard of care session of HTC, guided by Vietnamese and international guidelines [35].

Participants randomized to the individual level intervention arm received: 1) two individual posttest counseling sessions, in addition to standard of care HTC, that included discussions about coping with stigma, social support, partner testing, and disclosure; 2) two small group sessions consisting of 6–10 participants conducted by a team of two facilitators that focused on HIV knowledge and skill-building while simultaneously providing social support through shared experiences of being an HIV-infected PWID. Participants in the intervention arm were also offered an optional dyad session with a “person important to me” (PIM) to address how the self-identified PIM could best support the participant in coping with HIV and reducing HIV risk behaviors.

#### Structural level intervention

Community members from sub-districts randomized to the control arm received standard messages on HIV through village weekly public loudspeakers and educational pamphlets that were already being provided by community health stations.

Community members in sub-districts randomized to the intervention arm were invited to participate in a community-wide program consisting of a 2-part video and a series of 6 HIV education sessions delivered by a trained community mobilizer. These activities aimed to reduce community HIV and injecting drug use (IDU)-related stigma by correcting misconceptions about HIV transmission, de-linking PLWHA from “social evils” and promoting positive messages on HIV and PLWHA in the community. The video, entitled “The Traveling Firefly” was a fictional account of a boy whose mother was HIV-infected and the effect that primary and secondary stigma had on their family. The video script was based on extensive formative research conducted prior to the trial and directed and produced by the Johns Hopkins University’s Center for Communication. Times, dates and locations of video showings were selected based on formative research. Timing, frequency, delivery and content of educational sessions were also based on feedback during the formative phase. Sessions focused on dispelling misconceptions about HIV, PLWHA, and PWID. A team of community mobilizers recruited from the Women’s Union, a grass roots country-wide organization in Vietnam, was trained to lead the educational sessions during a 5-day centralized training in Thai Nguyen City. The video and educational sessions were continuously component tested with focus groups of community members in the province during development and a final pilot was conducted prior to use.

Video screenings and educational sessions were implemented after enrollment in the following manner. *Video*: One week before each of the four screenings, community members were invited to attend via daily public loudspeaker announcements and door-to-door visits. Attendance per screening was not restricted and community members could attend multiple screenings. Each video screening was followed by a question-answer session with a trained facilitator.

*Educational sessions*: Community mobilization volunteers in each intervention sub-district disseminated HIV information and answered questions through 6 rounds of a combination of one-on-one and small group discussions (attendance was open) in homes of willing community participants. Sessions occurred approximately every two months after the video screenings.

### Measures and outcomes

At every visit, index participants reported their education, marital status, employment status, sexual risk behaviors, injecting behaviors, internalized, perceived and experienced stigma, HIV knowledge, depression, disclosure of HIV status, partner uptake of HTC and ART and social support.

#### Risk assessment

Injecting and sexual risk were assessed at each visit. Participants were asked about direct (gave or received used needles/syringes) or indirect sharing (shared injecting drugs, solutions or distilled water) in the past 3 months. Frequency of injecting, defined as the number of days injected in the past 3 months and number of times injected per day were also assessed. For sexual risk, participants were asked if they ever had sex with a female or male sexual partner without using a condom in the previous 3 months.

#### HIV and IDU related stigma

Separate sets of items were used to assess HIV and IDU stigma. Participants were asked to respond to a statement on a 4-point Likert scale (strongly agree, agree, disagree, strongly disagree). For HIV-related stigma, 22 items were initially entered into principal components analysis. Maximum likelihood method of factor analysis was then applied for 3, 4, and 5 factors, using promax (i.e., oblique) rotation. Given that there was little qualitative difference between the items retained in the three different models, the parsimonious 3 factor model was chosen. The sum total of values of the 14 items from the 3-factor model formed the HIV-related stigma scale. The same methods were applied to construct the IDU-related stigma scale. Of the 13 items initially entered into the factor analysis for IDU-related stigma, 7 items, comprising 3 factors, were retained. The values of these items were summed to form the IDU-related stigma scale.

#### Social support scales

Social support was measured at every visit using the medical outcomes study (MOS) social support scale [36] and included four subscales as defined by Sherbourne: 1) emotional/informational support, 2) tangible support, 3) positive social interaction, and 4) affectionate support. Scores on these subscales ranged from 0–100.

#### Injecting network size

Participants were asked at every visit about injecting partners and injecting network size from the last 3 months. Injecting partners were defined as someone who was in the same room as or in close proximity to the participant when they were both injecting drugs. Injecting network size was defined as the total number of injecting partners listed by each participant.

### Statistical analysis

For each of the primary outcomes (any direct/indirect sharing and any unprotected sex) and secondary outcomes (HIV incidence among network members, stigma, social support, and size of injecting network), the first, main goal was to estimate the average of all available values under each of the four arms as assigned, and then compare across arms.

Because death rates differed by arm and visit (arm 2 visit 5 had lowest survival), and, among survivors, some visits were missed, a second goal was to estimate the average outcomes for a group of participants whose covariate profile is common across arms. Specifically, for each arm A = a, and time of visit t, we estimated the average outcome *Y*_t_ for participants who match the survivors of arm 2 and visit 5 in the distribution of all baseline covariates X; this quantity is expressed as

$$\int E\left(Y_{t}\;|\;A=a,S_{t}=1,R_{t}=1,X_{0}\right)\;\times \;pr\left(X_{0}\;|\;A=2,S_{5}=1\right)dX_{0}$$

where *S*_t_ = 1 indicates survival at time t and *R*_t_ = 1 indicates that the participant provided the outcome at time t. This quantity focuses on a common profile of individuals, so that any differences in a comparison across arms cannot be attributed to differential mortality or differential missed visits as far as can be predicted by covariates. We estimated the above quantity by empirically estimating

$$\sum_{e^{*}=1}^{5}E\left(Y_{t}\;|\;A=a,S_{t}=1,R_{t}=1,\;e_{a,t}^{*}\right)\cdot pr\left(e_{a,t}^{*}\;|\;A=2,S_{5}=1\right)$$

where *e* ^*^_a,*t*_ are the quantiles of the propensity score *pr*[*S*_t_ = 1, *A* = *a*, *R*_t_ = 1 | {(*S*_t_ = 1, *A* = *a*, *R*_t_ = 1) or (*S*_5_ = 1, *A* = 2)}, X_0_]. This adjustment is known as ‘stratification by quintiles of propensity score’ [37], and it is mostly used for calibrating between pre-treatment defined groups. It has also been shown to be useful for calibrating between groups defined post-treatment (e.g., in sequential designs [38]) and adjusts for the known variables for which the comparison groups differ. Finally, covariate calibration has also been used for matched-clustered designs as in Wu et al. [39], where it has been shown superior to alternative methods.

For both goals, standard errors of the estimates account for the matched district-level assignment of the structural intervention.

## Results

### Enrollment and retention

We enrolled 455 index participants and 355 HIV negative network members for a total of 810 participants. Among our index participants, 89 were in the control arm, 139 in the structural intervention arm, 95 in the individual intervention arm and 132 were in the individual and structural level arm combined (Fig 1). The different sample size in each arm was due to our two-stage randomization strategy. In the first stage we randomized by sub-district to the community intervention arm or the community control arm. In the second stage, we randomized participants in each sub-district to the individual intervention or the individual control arm, resulting in 4 arms. Because the number of HIV-infected PWID within each sub-district varied, the sample size for each arm was different. One hundred index participants did not choose to bring in a network member. Of the 1739 PWID who were initially screened, 1676 were deemed eligible to complete the baseline survey and HIV test, and 521 tested positive (HIV prevalence rate = 31%). Of those, 57 were not eligible for other reasons. Among those eligible, 9 declined to participate and 455 (98% response rate among eligible individuals) enrolled in the study. Among those eligible, those enrolled were more likely than non-enrolled to have ever been tested for HIV at baseline and were less likely than enrolled to inject daily in past 3 months at baseline. Those individuals enrolled were not statistically different from those not enrolled by socio-demographic characteristics or sexual risk behaviors. The overall retention rate at 24 months excluding those who died or were incarcerated was 94% among indexes and 79% among network members. Study retention was not related to treatment condition.

### Baseline characteristics

Table 1 characterizes the index participants by assigned arm. At baseline, participants were on average aged 35 years (range 19–60). About half of participants were married and most (70%) worked full-time. At baseline, self-reported direct and indirect sharing was the norm, with approximately 26% having shared needles/syringes and 70% having shared equipment/solution in the past 3 months. About a quarter reported having any unprotected sex in the past 3 months. Forty percent had ever been tested for HIV prior to baseline visit, 36% had ever been incarcerated, and 75% felt they had been stigmatized in their community due to drug use. The mean CD4 count was 272 (SD = 182). No participants reported sex with a male partner at baseline. Accounting for clustering within matched sub-district groups, there were no significant differences between arms with respect to demographic or risk characteristics.

**Table 1:** Baseline characteristics of enrolled index participants (N = 455), by trial arm.

Baseline Characteristic	Total N = 455	Control n = 89	Community Intervention Only n = 139	Individual Intervention Only n = 95	Combined Intervention n = 132	p-value*
***Age*, *in years*:**						
Mean (s.d.)	35.2 (6.3)	33.7 (5.0)	35.4 (6.4)	35.7 (6.5)	35.7 (6.7)	0.12
***Years of education*:**						
Mean (s.d.)	8.6 (2.9)	8.1 (3.3)	8.9 (2.8)	8.5 (2.5)	8.5 (3.1)	0.44
***Marital status*: *%***						
Single (never married)	38	37	44	31	39	0.51
Married	47	53	44	49	45	
Widowed/ divorced/ separated	14	10	12	20	15	
***Employment status*: *%***						
Work full-time (≥30 h/w)	70	73	65	75	67	0.53
Work part-time (<30 h/w)	18	11	21	18	20	
Unemployed/unable to work/other	13	16	14	7	13	
***Income per month (Vietnamese Dong*, *in millions)*:**						
Mean (s.d.)	1.8 (1.2)	1.6 (0.9)	1.7 (1.1)	1.9 (1.4)	1.9 (1.2)	0.12
***Religion*: *%***						
No religion	96	98	94	99	95	0.49
***Ethnic group*: *%***						
Kinh (ethnic Vietnamese)	87	84	86	89	86	0.27
***Have you ever spent a night on the street*, *in a park*, *alley*, *or abandoned building*, *in the last 3 months*?**						
% Yes	12	9	15	8	14	0.71
***% Who had any sharing*, *past 3 months***	73	75	76	61	77	0.14
***% Who had any direct sharing (needle/syringe)*, *past 3 months***	26	27	27	20	29	0.39
***% Who had any indirect sharing (other injecting equipment/ liquids)*, *past 3 months***	71	72	75	60	76	0.43
***% Who injected daily*, *past 3 months***	54	40	48	43	48	0.48
***% Who ever overdosed on drugs in lifetime*?**	18	16	24	16	15	0.47
***% Who were ever in drug treatment in lifetime***	31	24	33	29	36	0.54
***% Who had any unprotected sex*, *past 3 months***	24	21	22	23	27	0.60
***Number of different female sex partners*, *past 3 months*:**						
Mean (s.d.)	0.71 (0.94)	0.60 (0.56)	0.64 (0.83)	0.80 (1.06)	0.81 (1.13)	0.34
***% Who had ever been tested for HIV prior to baseline visit***	41	47	40	43	36	0.84
***In general*, *how would you say your health is*?**						
% Poor health	30	34	32	23	30	0.67
***% Who have ever been incarcerated (put in prison*, *jail*, *or detention center) in lifetime***	36	34	43	27	35	0.09
***In your opinion*, *are you stigmatized by your community because of your drug use*?**						
% Yes	75	74	75	74	76	0.98
**CD4 count:**						
Mean (s.d.)	272 (182)	264 (161)	254 (188)	277 (176)	292 (193)	0.12

*P-values are based on Wald Chi-squares with 3 df, using GEE with independent correlation structure, clustering on sub-district matched groups.

### Intervention Exposure

Among 227 participants randomized to the individual level intervention, 93%, 92%, 86% and 85% attended individual sessions 1 and 2, and group sessions 1 and 2 respectively. Eighty-three percent attended all 4 sessions, and 67% attended an optional session with a support person (Person Important to Me or PIM). Two-hundred and seventy one participants were randomized to the structural level intervention. Ninety percent of the subjects in the community member cohort attended the first video screening, and 82% attended the second video screening. An additional 1919 and 2164 non-enrolled community members attended video sessions 1 and 2, respectively. Ninety-seven percent of subjects in the community member cohort, who were present at home when approached by the Women’s Union representatives, agreed to participate in all 6 rounds of door-to-door sessions with women from the Women’s Union.

### Intervention Effect

#### HIV incidence

Among 270 network members with 572 person-years of follow-up, there were 4 seroconversions. The incidence rates were as follows: Arm 1: 10/1000 person years (py); Arm 2: 5/1000 py; Arm 3: 18/1000 py; and Arm 4: 0/1000 py) (Table 2). There was no significant difference in seroconversions between intervention and control arms over a 24-month period (Cox-regression p-value = 0.261).

**Table 2:** HIV incidence overall and by arm.

	No. Network Members	No. HIV seroconversions	Total person-years	HIV incidence rate per 1000 py
Overall	355	4	572.29	7
Control	62	1	95.54	10
Community intervention only	114	1	194.39	5
Individual intervention only	69	2	110.23	18
Combined intervention	110	0	172.14	0

#### Direct/indirect sharing

Rates of reported sharing among indexes decreased gradually from 77% at baseline to 4% at 24 months (p< 0.001) (Table 3). Sharing at 24 months was comparable among arms (Wald = 3.40 (3df); p = 0.33), and this result was robust when rates were calibrated for survival and attended visits as described in the statistical analysis section.

**Table 3:** Percent and standard error (se) of index participants who had shared any injecting needles, syringes or equipment in the past 3 months.

Visit No.	Baseline	6-Month	12-Month	18-Month	24-Month
**Control**					
*(No*. *observed)*	*(89)*	*(75)*	*(68)*	*(60)*	*(64)*
Observed data: %(se)	75 (5)	19 (5)	13 (4)	2 (2)	2 (2)
Calibrated: %(se)	79 (9)	18 (10)	12 (10)	1 (7)	2 (6)
**Community Intervention Only**					
*(No*. *observed)*	*(139)*	*(113)*	*(91)*	*(80)*	*(78)*
Observed data: %(se)	76 (3)	16 (3)	12 (2)	6 (2)	5 (3)
Calibrated: %(se)	76 (3)	16 (3)	13 (2)	7 (3)	5 (4)
**Individual Intervention Only**					
*(No*. *observed)*	*(95)*	*(76)*	*(62)*	*(56)*	*(58)*
Observed data: %(se)	61 (7)	20 (6)	13 (7)	7 (6)	3 (2)
Calibrated: %(se)	77(11)	23 (11)	16 (12)	9 (16)	8 (9)
**Combined Intervention**					
*(No*. *observed)*	*(132)*	*(113)*	*(106)*	*(96)*	*(97)*
Observed data: %(se)	77 (4)	12 (4)	8 (3)	10 (3)	2 (1)
Calibrated: %(se)	76 (6)	12 (6)	9 (5)	14 (5)	3 (2)

#### Unprotected sex

Fewer participants also reported unprotected sex, from 24% at baseline to an average of 5% at 6 months (p<0.001) and remained as low during follow-up (Table 4). The observed rates of unprotected sex were not significantly different at the 24-month visit among the four arms (Wald = 6.73 (3df); p = 0.08) although the observed rates were lower for the arms with the single interventions. These results were also robust when rates were calibrated for survival and attended visits.

**Table 4:** Percent and standard error (se) of index participants who had unprotected sex in the past 3 months.

Visit No.	Baseline	6-Month	12-Month	18-Month	24-Month
**Control**					
*(No*. *observed)*	*(89)*	*(75)*	*(68)*	*(60)*	*(64)*
Observed data: %(se)	21 (9)	4 (3)	3 (3)	2 (2)	6 (4)
Calibrated: %(se)	23 (11)	5 (8)	5(6)	1 (6)	11 (10)
**Community Intervention Only**					
*(No*. *observed)*	*(139)*	*(113)*	*(91)*	*(80)*	*(78)*
Observed data: %(se)	22 (3)	3 (1)	5 (2)	3 (2)	1 (1)
Calibrated: %(se)	22 (3)	3 (1)	5 (2)	2 (3)	1 (1)
**Individual Intervention Only**					
*(No*. *observed)*	*(95)*	*(76)*	*(62)*	*(56)*	*(58)*
Observed data: %(se)	23 (6)	4 (3)	2 (2)	4 (3)	0 (0)
Calibrated: %(se)	27 (7)	1 (3)	0 (1)	0 (1)	0 (0)
**Combined Intervention**					
*(No*. *observed)*	*(132)*	*(113)*	*(106)*	*(96)*	*(97)*
Observed data: %(se)	27 (5)	8 (2)	4 (1)	4 (3)	3 (2)
Calibrated: %(se)	22 (8)	7 (2)	5 (3)	4 (5)	5 (3)

#### HIV/IDU stigma

Average HIV stigma scores remained stable over the follow-up and comparable across arms (Table 5).

**Table 5:** Mean and standard error (se) of HIV stigma score among index participants.

Visit No.	Baseline	6-Month	12-Month	18-Month	24-Month
**Control**					
*(No*. *observed)*	*(89)*	*(75)*	*(68)*	*(60)*	*(64)*
Observed data: mean (se)	29.9 (0.4)	28.5 (0.7)	28.4 (0.5)	28.6 (0.5)	29.3 (0.7)
Calibrated: mean (se)	29.1 (0.9)	28.6 (1.0)	28.3 (0.6)	28.2 (1.6)	29.9 (1.0)
**Community Intervention Only**					
*(No*. *observed)*	*(139)*	*(113)*	*(91)*	*(80)*	*(78)*
Observed data: mean (se)	28.9 (0.7)	28.5 (0.6)	28.8 (0.4)	29.3 (0.3)	28.8 (0.4)
Calibrated: mean (se)	28.9 (0.7)	28.6 (0.6)	28.8 (0.3)	29.4 (0.4)	28.8 (0.3)
**Individual Intervention Only**					
*(No*. *observed)*	*(95)*	*(76)*	*(62)*	*(56)*	*(58)*
Observed data: mean (se)	29.7 (0.2)	28.7 (0.4)	28.9 (0.3)	28.6 (0.3)	28.1 (0.4)
Calibrated: mean (se)	28.9 (1.3)	28.1 (0.5)	28.8 (0.8)	28.8 (0.8)	28.1 (0.9)
**Combined Intervention**					
*(No*. *observed)*	*(132)*	*(113)*	*(106)*	*(96)*	*(97)*
Observed data: mean (se)	30.3 (0.5)	28.7 (0.6)	28.5 (0.3)	28.7 (0.3)	28.9 (0.3)
Calibrated: mean (se)	29.3 (1.0)	28.3 (0.4)	28.1 (0.4)	28.2 (0.6)	28.4 (0.6)

IDU stigma decreased by an average of 0.6 units (20% of a standard deviation) between baseline and 24 months (se = 0.20, p = 0.002), although this result was not quite reproducible with the calibrated analysis (p = 0.063). IDU stigma at 24 months was comparable across arms (Table 6).

**Table 6:** Mean and standard error (se) of IDU stigma score among index participants.

Visit No.	Baseline	6-Month	12-Month	18-Month	24-Month
**Control**					
*(No*. *observed)*	*(89)*	*(75)*	*(68)*	*(60)*	*(64)*
Observed data: mean (se)	18.4 (0.3)	17.9 (0.4)	17.7 (0.4)	18.6 (0.4)	18.3 (0.6)
Calibrated: mean (se)	19.1 (0.4)	18.3 (0.4)	17.3 (0.6)	18.2 (0.6)	18.7 (0.8)
**Community Intervention Only**					
*(No*. *observed)*	*(139)*	*(113)*	*(91)*	*(80)*	*(78)*
Observed data: mean (se)	19.2 (0.3)	18.1 (0.2)	18.4 (0.2)	18.6 (0.3)	18.2 (0.3)
Calibrated: mean (se)	19.2 (0.3)	18.1 (0.2)	18.5 (0.3)	18.7 (0.3)	18.2 (0.3)
**Individual Intervention Only**					
*(No*. *observed)*	*(95)*	*(76)*	*(62)*	*(56)*	*(58)*
Observed data: mean (se)	18.1 (0.3)	18.0 (0.2)	17.6 (0.1)	17.6 (0.3)	17.9 (0.3)
Calibrated: mean (se)	19.3 (0.7)	18.2 (0.7)	18.2 (0.5)	17.2 (0.9)	18.0 (1.0)
**Combined Intervention**					
*(No*. *observed)*	*(132)*	*(113)*	*(106)*	*(96)*	*(97)*
Observed data: mean (se)	18.9 (0.3)	17.7 (0.3)	17.8 (0.4)	17.9 (0.2)	17.8 (0.2)
Calibrated: mean (se)	18.7 (0.6)	17.7 (0.7)	17.8 (0.4)	18.0 (0.3)	17.7 (0.3)

#### Other proximal outcomes

One month after HIV diagnosis through HTC, approximately 70% of participants in all arms said they had disclosed their status to at least one person. This number reached close to 90% at 24 months follow-up. No difference was observed across arms (data not shown). Emotional/informational social support increased over time in all arms except the combined intervention arm. All other social support scales remained about the same over time across all arms. The mean number of people in injecting networks decreased over time in all arms (baseline to 24 months: 1.7 (SD = 1.1) to 0.1 (SD = 0.4) persons in the control arm; 1.9 (SD = 1.3) to 0.3 (SD = 0.7) persons in the community level intervention only arm; 1.6 (SD = 1.2) to 0.2 (SD = 0.5) in the individual intervention only arm; and 2.0 (SD = 1.6) to 0.1 (SD = 0.5) in the combined intervention arm).

## Discussion

In our study, participants in all four arms dramatically reduced their injecting and sexual risk behaviors between baseline and 6 months, and at 24-month follow-up there was no difference across arms. Incidence rates among HIV negative networks members were low (Control: 10/1000 person years (py); Community intervention only: 5/1000 py; Individual intervention only: 18/1000 py; and Combined intervention: 0/1000 py) with no difference across arms (Cox regression p-value = 0.261). Previous studies have found an incidence rate of 50/1000 in Thai Nguyen [31] and 200/1000 py in Long San Province, a northern Province in Vietnam bordering China [40], compared to 10/1000 py in the control arm in our study. Understanding the mechanisms through which all arms, particularly the control arm, demonstrated both low risk behaviors and low HIV incidence has important implications for policy and prevention programming.

Other RCTs of individual level HIV risk reduction behavioral interventions among both PWID and the general population, including our previous study in Vietnam, have observed decreases in HIV risk behaviors or disease outcomes across treatment and control arms [31, 41, 42]. Possible explanations for our findings include the effectiveness of the standard of care program, social desirability bias and contamination between the intervention and control arms.

HTC is a critical component of treatment, care, and prevention efforts [43], and has been widely implemented in industrialized and developing countries [44, 45]. Research has shown that HTC can change HIV-related risk behaviors particularly among HIV-infected participants, confirming its importance as an HIV prevention strategy [28]. Among PWID, knowing one’s status has been associated with a reduction in risky behaviors. For example, in a study conducted in Ukraine, HIV-infected PWID who knew their status were significantly more likely to use condoms consistently with a non-injecting permanent partner compared to those who did not [46]. Assessment visits that offer access to an effective standard of care delivered by a well-trained study staff have been cited as a possible explanation for why RCTs may not show a significant difference in the reduction of behavioral risk [42, 47]. All study participants received pre and post-test HIV counseling from counselors who were intensively trained on the WHO protocol and sensitized to working with substance users and treating them with respect and dignity.

In our study, 68% of participants said they did not know their HIV status prior to our study. Those who knew their status prior to our study and were HIV-infected were, at baseline, significantly less likely to engage in direct or indirect sharing and less likely to engage in unprotected sex in the past 3 months than participants who did not know their status. Those who knew their status and were HIV-negative were not significantly different in HIV risk behavior compared to those who did not know their status. This suggests that discovering one’s HIV-infected status in concert with pre- and post-test counseling delivered by fully trained counselors may have contributed to the marked change in injecting and sexual behaviors. Qualitatively, we have found among newly diagnosed HIV-infected participants that protecting family and support networks is of paramount importance [19, 21]. Forward transmission was voiced as a primary concern, to the degree that some participants moved out of their homes to protect their families. In a family and community centered culture like Vietnam’s, participants’ discovery of HIV status through HTC and the resulting concern about forward transmission may be especially powerful and may explain, in part, the dramatic reductions we saw in HIV risk behaviors among participants in all four arms. Interestingly, we compared the results of those who knew their status at baseline compared to those who did not know their status and found that even for participants who knew their status at baseline, there was qualitatively similar reduction in risky behaviors. This suggests that, in addition to knowledge of status itself, there were other factors that reduced self-reported risk behaviors.

In addition to HTC, several evidence-based programs were in place throughout Thai Nguyen during the period in which this study was conducted, which could also have provided support to risk reduction efforts. These included multiple needle exchange programs as well as a first methadone maintenance therapy clinic, which opened in Thai Nguyen in 2011. In addition, free condoms were available at the project site. It may be, as has been previously shown, that HTC as part of a combination prevention package may lead to significant reductions in risk behaviors [25, 48].

Positive responses may have been influenced by fear of incarceration and/or detention. In Vietnam, when individuals are suspected of engaging in criminal activities such as theft or drug trafficking, they are placed in a police station, or jail and, if convicted, in prison. In contrast, individuals who police suspect of engaging in injecting drug use and who are then subject to urine drug testing, are confined in drug treatment centers for typically about a year if results of that testing are positive. Our team took several measures to assure individuals that participation in the study was completely voluntary, completely confidential and would not be cause for incarceration and/or detention. In the ten years that we have been working with PWID in Thai Nguyen, there have been no arrests related to research participation among our participants.

Social desirability bias may also have contributed to our results. Our primary and secondary outcomes were based on self-report and in Vietnam, where family, social and community approval and acceptance are highly valued [21, 49], the desire to report socially acceptable behavior may be particularly strong. Further, since an HIV-infected status may heighten perceptions of disapproval for putting others at risk, social desirability may be more likely in this study.

However, our biological endpoint, HIV incidence, supports the validity of our self-reported risk behaviors. Specifically, our previous study in Thai Nguyen found an incidence rate of 5% among HIV negative PWID [31]. We would expect a similar or higher HIV incidence rate between discordant injecting partners. The lower than expected HIV incidence rate among network members in our study is consistent with the low self-reported injecting risk behaviors. However, future studies should examine or other biological outcomes to minimize the potential for social desirability bias. In addition, further research is needed to explore the psychological and emotional context through which PWID may respond to interviewers in a longitudinal trial.

Finally, contamination between the intervention and control arms could also have had an impact on our findings. To examine the possibility of message diffusion from the intervention to control, we asked our control participants at each visit, how many friends who inject drugs had talked to them about how to prevent HIV through safe injecting practices; if contamination occurred, we would expect to see an increase in conversations initiated by an injecting friend over time. Based on our survey data, the control arm did not increase the number of HIV conversations, suggesting that contamination across arms may not have occurred.

Despite the significant reduction in HIV risk behaviors (our primary outcomes) across all arms, one of our hypothesized mediators, stigma, did not change over time in any of the four arms. This finding may in part be due to an issue with measurement. We employed ICRW’s stigma scales that have been validated in the Vietnamese context, used in many settings in Vietnam and globally [50]. Despite the strength of these scales, however, the items in the scale did not capture a main goal of our intervention: *coping* with stigma. In addition, the vast majority of responses for each of the items were in the range of 2–3 on the scale (e.g., 90% of respondents answered 2 or 3 on the scale of 1–4 to the statement, “Families of people living with HIV/AIDS should be ashamed.”), making it difficult to discern any potential changes in stigma.

This study had several limitations. The study was conducted in a context where study conditions could not be tightly controlled for self-reporting bias, attrition, secular trends, historic factors and contamination. Our previous RCT investigated the possibility of these conditions and did not find evidence that they influenced results [31]. However, the possibility of these factors operating cannot be ruled out. Another limitation to this study was that HIV was the only biological marker that we were able to use. Based on our previous work, the prevalence of bacterial STDs, including chlamydia, gonorrhea and syphilis in this population was too low (9%, 0 and 1% [3]) and prevalence of HBV and HCV too high (80.9, 74.1 [51]) to use as biomarkers, since changes in incidence of these infections would be difficult to detect. Finally, the sample was not randomly selected, which limits the generalizability of the findings.

Despite these limitations, our study suggests that multi-level interventions are acceptable by both PWID and non-PWID community members. The intervention was well attended by both index PWID and non-PWID community members, with 83% and 78% attending all sessions at each level, respectively. Our results suggest that HTC provided by carefully trained counselors and in a community context that supports risk reduction through programs such as needle exchange and MMT may be very effective in reaching HIV infected PWID and strengthening their risk reduction efforts. These findings may have broad relevance to other epidemics, such as those in Sub-Saharan Africa, and especially in settings where communities are well defined and where social interactions are frequent and impactful.

## Supporting Information

S1 CONSORT Checklist CONSORT checklist. (PDF)

S1 Dataset (CSV)

S1 Protocol Trial protocol. (PDF)

S2 Protocol Vietnamese translation of the trial protocol. (PDF)

S1 Table Percent and standard error (se) of index participants who had shared any injecting needles, syringes or equipment in the past 3 months, stratified by knowledge of HIV status at baseline. (DOCX)

S2 Table Percent and standard error (se) of index participants who had unprotected sex in the past 3 months, stratified by knowledge of HIV status at baseline. (DOCX)
