# Supplementary material for: Efficacy of a Multi-level Intervention to Reduce Injecting and Sexual Risk Behaviors among HIV-Infected People Who Inject Drugs in Vietnam: A Four-Arm Randomized Controlled Trial
Source: PLoS One. 2015 May 26;10(5):e0125909. doi: 10.1371/journal.pone.0125909 (PMC4444299; doi:10.1371/journal.pone.0125909)
Supplement: S1 Protocol — (PDF) [file pone.0125909.s003.pdf]

RESEARCH PLAN

PI: Vivian F. Go, PhD

Study Title: Prevention for Positives: A Randomized Controlled Trial Among Vietnamese HIV-positive IDUs

Sponsor's Protocol # *IRB00000594*

Research Question:

This study is a randomized controlled trial among HIV-positive injecting drug users (IDUs) to reduce high risk injecting and sexual behaviors in Thai Nguyen, Vietnam. We will be conducting this study in collaboration with the Thai Nguyen Centre for Preventive Medicine (CPM). We will compare standard HIV voluntary counseling and testing (VCT) to an intervention that adds a continuum of psychosocial support for HIV-positive IDUs. Our intervention includes community-based stigma reduction programs, and will assess both individual and structural interventions. Our research questions for the overall project are:

1. What is the impact of perceived HIV-related stigma on disclosure, social support and coping among IDUs?
2. What roles do disclosure, social support and coping play in facilitating the reduction of HIV-risk behaviors
3. Are combined individual and structural level interventions more effective in reducing injecting and sexual behaviors among HIV-positive IDUs than individual and structural interventions alone and more effective than the standard HIV VCT?

Hypothesis

Our primary hypothesis is that the combined individual and structural level activities is effective in reducing injecting and sexual behaviors. Our secondary hypothesis is that network members of IDUs assigned the individual and structural level interventions will have decreased HIV incidence after 24 months.

Rationale:

Background

HIV-positive IDUs occupy a critical place among HIV-positive risk groups because they can potentially transmit the HIV virus through two routes: unprotected sex and unsafe drug use (1). In Vietnam, IDUs account for over 65% of all reported HIV infections, and HIV sentinel surveillance indicates that HIV continues to increase in all surveillance groups, including IDUs (2). With rising HIV prevalence, increasing IDUs and female sex worker population who use drugs, IDUs may soon influence HIV risk in the general population. Interventions that enhance HIV knowledge, facilitate voluntary disclosure of HIV status and strengthen social support among HIV-positive persons may reduce sexual and injecting risk behaviors, which in turn, reduce HIV transmission (3-6).

HIV-related stigma has been widely reported in Vietnam, and is one of the main obstacles to prevention, care and treatment of HIV/AIDS (7-9). People living with HIV (PLWH) in Vietnam

often experience severe social marginalization within families, communities, schools and in the workplace, discrimination in health care settings, and internalized stigma (7, 10). As a result, stigma prevents disclosure of HIV status to others and is a profound barrier to HIV prevention, access and support in Vietnam.

Aims

The specific aims of this project are:

1. To qualitatively explore the impact of perceived HIV-related stigma on disclosure, social support and coping among IDUs, and to explore, in turn, the role of disclosure, social support and coping in facilitating the reduction of HIV risk behaviors among HIV-positive IDUs in Thai Nguyen, Vietnam.
2. To conduct a four-arm randomized attention-controlled intervention trial among HIV-positive IDUs. The four arms of the trial are: 1) an attention-controlled condition; 2) individual-level posttest counseling and skill-building support groups; 3) structural-level stigma reduction programs; and 4) both individual and structural level activities.

Methods:

Study Design and Rationale:

Phase I: Formative:

Formative ethnographic research will be used to explore the impact of perceived HIV-related stigma on disclosure, social support, and coping among IDUs and to explore the role of disclosure, social support, and coping in facilitating the reduction of HIV risk behaviors among HIV-positive IDU to help inform the intervention and refine measures used in the survey instrument. To achieve this, we will conduct qualitative interviews with the following goals:

1.) Explore the impact of HIV-related stigma on disclosure, social support and coping and to explore the role of stigma, social support and coping in facilitating the reduction of HIV risk behaviors among HIV-positive IDUs

2.) Describe women's experiences with their partner's HIV-serostatus disclosure

3.) Describe community attitudes and behaviors towards HIV-positive individuals and towards the Women's Union and Youth Union

4.) Conduct a pilot of assessment measures

5.) Conduct a pilot of components of the intervention.

Phase II-IV: Baseline, Intervention and Evaluation

To determine the effects of combined and separate individual and structural level activities on injecting and sexual behaviors, a four arm randomized intervention trial will be conducted. Two villages will be randomly selected from each of the 4 districts in Thai Nguyen with the largest numbers of IDUs. Within each district, one village will be randomly assigned to receive the stigma reduction programs and the other to receive the control, and within each village (regardless of stigma reduction assignment) a random half of the Index IDUs will be assigned to the intervention (enhanced posttest counseling and skill building support groups) and the other half to the control. The overall study design is shown below in Figure 1. The primary outcomes among

Index participants will be drug-related and sexual risk behaviors. The primary outcome among Network participants will be HIV incidence. The intermediate outcomes will include perceived stigma, correct condom use, partner uptake of HIV VCT, perceived social support, depression, and self-reported disclosure.

Figure 1. Study Design

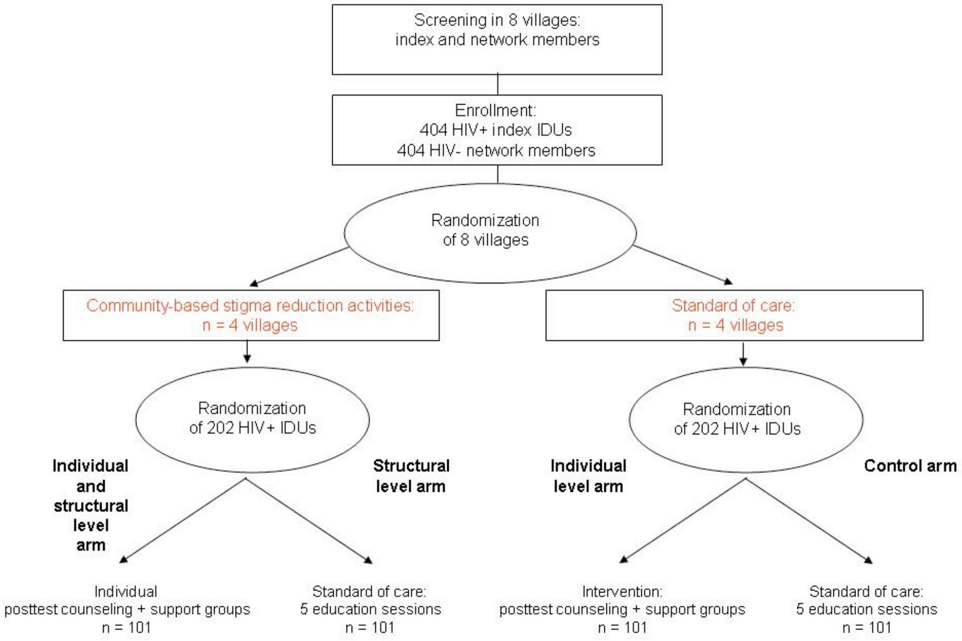

Among participants who are randomized to the intervention arm and who return for their follow-up assessments, we will conduct qualitative interviews among 10 participants at each follow-up visit. The purpose is to explore participants' experiences with injecting and sexual behavior changes and HIV-related stigma.

To assess operational outcomes across study arms, we will compare participants' perceived quality of sessions, proportion of IDUs attending all posttest counseling sessions and cost of service provision and opportunity cost incurred by participants. We will use ongoing in-depth interviews with a subset of our participants, analysis of project records, including participant evaluation forms, counseling and session log forms and financial records. Outcome evaluation will include pre-intervention assessment of participants with post-intervention follow-ups at 3, 6, 12, 18 and 24 months.

To assess HIV incidence among HIV-negative injecting network members of HIV-positive IDUs, injecting partners of HIV-positive IDUs enrolled in the intervention trial will be screened and tested for HIV status and a follow-up assessment will be conducted 24 months from the intervention start date of their respective index IDUs. Network members will be asked to provide a list of names of their injecting partners and their injecting practices with each partner over the past two years, enabling assessment of the possibility of network member's HIV exposure through injecting sources other than through the index participant.

Population:

Phase I: Formative

Sample Size:

Approximately 170 participants will be recruited for ethnographic interviews, including in-depth interviewees and focus group discussants. We will conduct 24 in-depth interviews with male HIV-positive IDUs and 10 in-depth interviews with female partners of IDUs in order to explore the impact of HIV-related stigma on disclosure, social support and coping and the roles of disclosure, social support and coping in facilitating the reduction of HIV risk behaviors, and to explore women's experiences with their partner's HIV-serostatus disclosure. We will also do two additional in-depth interviews with men of unknown HIV status and unknown drug using status so as avoid the possibility of the HIV-positive IDUS from being identified as such. Similarly we will interview 2 additional women of unknown partnership, HIV or IDU status for the same reason. We will conduct 2 focus group discussions among 8 men of unknown HIV and drug using status, 2 focus group discussions among 8 women of unknown HIV and drug using status, and 1 focus group discussion among community leaders to understand HIV-related stigma and to assess community attitudes towards the Women's and Youth Unions. We will also conduct 4 focus group discussions of 6-8 men and women in the community to pilot components of the community-level intervention, 6 focus group discussions among 6-8 male HIV-positive IDUs to pilot components of the individual-level intervention. In addition, we will pilot assessment measures among 20 HIV-positive male IDUs.

Power Calculations or Statistical Plan:

Because qualitative research stresses depth more than breadth and insight more than generalization, sample sizes are relatively small compared to survey research. The sample size depends on the number of people needed to adequately answer the research question.

Inclusion and Exclusion Criteria:

Individuals to be selected for all qualitative interviews will: 1) be 18 years of age or older; 2) reside in Thai Nguyen province and 3) be capable of providing voluntary informed consent. Individuals will be excluded if they 1) are younger than 18 years of age; 2) reside in communities outside of Thai Nguyen province; or 3) are incapable of providing voluntary informed consent.

Individuals selected for in-depth interviews on the impact of HIV-relative stigma also will 1.) be male; 2.) be an HIV-positive current or former injection drug user who was screened in our former study but was not enrolled due to their HIV seropositive status; 3) have indicated willingness to be contacted for future study; 4) have known his HIV status for approximately 1 year.

Individuals selected for in-depth interviews on women's experiences with their partners HIV-serostatus disclosure also will 1) be female; and 2) be a sexual partner of an HIV-positive IDU.

Individuals selected for piloting assessment measures or for piloting individual-level intervention components also will: 1.) be HIV-positive injection drug users.

Gender, Age and Locale

Based on our-HIV-negative study, the vast majority of IDUs in Thai Nguyen are male (97%), are heterosexual (none reported sex with another man) and in their mid-to-late twenties. We've restricted participation to those 18 years and older because under Vietnamese regulations, individuals 18 years and older can independently consent to participate in a research study without the consent of a parent.

In-depth interviews and focus group discussions will be conducted at a private assessment venue located centrally in Thai Nguyen province.

Phase II-IV: Baseline, Intervention and Evaluation

Sample Size and Power Calculations

For our primary hypothesis, that Index IDUs assigned the individual and structural level interventions will have decreased frequency of sexual and injecting risk behaviors, a total sample size of 404 HIV-positive IDUs enables us to detect a 40% or 50% decrease in sexual and injection risks, accounting for a 20% drop-out rate. Power calculations assumed intention-to-treat distributions, 85% power and an alpha of .05. With a sample size of 404, we can detect decrease in frequency of unprotected sex of .40 or above (assuming the stigma reduction programs are as effective as the individual-level intervention; .50 or above if it's 50% as effective) for analyses on IDUs. If the variance covariance parameters are similar between the sexual and drug risk trajectories, we can also detect an increase in frequency of using new needles and syringes of .40 or above for analyses on IDUs.

For our secondary hypothesis, that network members of IDUs assigned the individual and structural level interventions will have decreased HIV incidence, we estimate that a sample size of 400 (200 combined sample size for the arm receiving both interventions and the arm receiving both controls) HIV-negative network members will enable us to assess differences in HIV incidence among the HIV-negative injecting network members of HIV-positive IDUs, accounting for a 20% drop-out rate (160 x 2/ (1-20/100)). With 80% power and alpha of .05, we can detect a decrease in 24-month HIV incidence of between 20 -25% or more for analyses on injecting network members.

Inclusion and Exclusion Criteria

To be eligible for participation in this study, individuals must meet the following criteria: 1) HIV-positive diagnosis confirmed through testing in our study; 2) time since first notification of HIV-positive status less than 30 days prior to interview; *3) able and willing to bring in an injecting network member for screening;* 4) being male; 5) 18 years of age or older; 6) had sex in the previous 6 months; 7) injected drugs in the previous 6 months; and 8) planned residence in Thai Nguyen for the next 24 months.

Exclusion criteria: 1) is unwilling to provide locator information; 2) is unable to participate in the group due to psychological disturbance, cognitive impairment or threatening behavior; or 3) is currently participating in other HIV or drug use intervention activities. At the screening visit, we will ask a series of questions about whether the participant has been previously tested for HIV, the approximate dates of the tests, and result of the most recent test. We will exclude females in this study because 97% of IDUs in Thai Nguyen are male and the vast majority of females who inject drugs are sex workers and would require a different intervention to address their unique risk factors. We will recruit newly diagnosed HIV-positive IDUs since IDUs who have known their status for a long time may differ in their mental and physical states than those who have just learned their diagnosis.

As part of the screening interview, index participants will be asked to provide a list of 10 names of their injecting networks. A network inventory will be performed shortly after the screening interview to identify the first three peers listed that meet the following network enrollment criteria: 1) injected or shared drug paraphernalia with index in the past 6 months; and 2) interacts at least once a week with the index. In order to participate in the intervention component of the study, the index will be required to bring an eligible injecting network member into the study. The index participant may either accompany a network member to the study site or provide them with a sealed envelope containing information about the study. Network members who present for screening will be verified through a database of names provided by index participants. Those who decide to continue with the study will be screened: Eligibility criteria for network members: 1) HIV-negative; 2) 18 years or older; 3) injected or shared drug paraphernalia with index in past 6 months; and 4) interacts at least once a week with index. Exclusion criteria: 1) unwilling to provide locator information; 2) has psychological or cognitive impairment; or 3) currently participating in other HIV interventions. Study staff will further describe study objectives, procedures, risks and benefits to eligible participants, and answer any questions. Informed consent will be obtained, and a study network participant card issued.

Gender, Age and Locale

We will exclude females in this study because 97% if IDUs in Thai Nguyen are male and the vast majority of females who inject drugs are sex workers and would require a different intervention to address their unique risks. We've restricted participation to those 18 years and older because under Vietnamese regulations, individuals 18 years and older can independently consent to participate in a research study without the consent of a parent.

Assessment and intervention sites are in separate locations a mile from each other within Thai Nugyen City to ensure that the assessment team is blinded to participants' intervention assignments. The assessment facility consists of 2 buildings with 7 private, soundproof rooms. Separate rooms are used to receive participants, administer interviews, and to conduct HIV counseling and to collect specimens. Every effort is made to ensure that both facilities are as anonymous as possible (e.g., no signs outside identifying the building), in order to protect participant confidentiality.

Procedures:

Phase I: Formative

Recruitment Process:

Participants for in-depth interviews will be identified using purposive sampling. Specifically, we will select 12 HIV-positive IDUs who have disclosed their HIV status to at least one person and 12 HIV positive IDUs who have not disclosed to anyone from HIV-positive IDUs who were screened in our previous study but were not enrolled due to their HIV seropositive status and who had indicated their willingness to be contacted for future studies (n ~ 280). We are purposively sampling IDUs who have known their status for 1 year in order to understand the initial impact of the HIV diagnosis on their mental health and injecting and sexual behaviors. We will also purposively sample HIV-positive IDUs for piloting our assessment measures and individual-level intervention components. The two women and two men of unknown HIV or drug using status will be recruited form the general community by the health staff in Thai Nguyen.

We will recruit 10 female partners for in-depth interviews through volunteer self referrals from male participants. Ethnographers will ask IDUs who report that they have shared their HIV test results with their partner whether they would be willing to refer their partner for an interview.

For focus group discussions about community attitudes and behaviors and piloting of community-level intervention components, we will recruit men and women of unknown HIV or drug using status from the general community through the community health staff in Thai Nguyen.

Interviewers will read and explain the informed consent process to eligible participants, and if the participant agrees, written consent will be obtained and documented. All subjects will be given a copy of the form. All project activities will occur in a private project facility in Thai Nguyen. To reimburse participants for their time, participants will be given the Vietnamese Dong equivalent of $6.50 for in-depth interviews and $6.50 for focus group discussions.

Study Procedures:

In-depth Interviews: Total participants will include 24 HIV-positive IDUs who have known their status for 1 year, 12 of whom have disclosed their status to at least one person, and 12 of whom who have not disclosed their status to anyone, as 10 female partners of HIV-positive IDUs. In depth interviews of HIV-positive IDUs who focus on how participants learned of their HIV-status, and their experiences with stigma, the extent and nature of their social support networks as well as their injecting and sexual risk practices over the past year. Ten in-depth interviews with female partners of HIV-positive IDUs will focus on the women's experience

with their partner's HIV serostatus disclosure. Interviews will be tape-recorded and will last approximately 1 hour. The four men and women of unknown status that will be interviewed will be asked similar questions to the formative phase focus group participants described below, including questions about community attitudes towards PLWH, towards the Women's Union and towards the Youth Union. The Women's Union and Youth Union are non-governmental voluntary grassroots organizations established in the early 1930s at national, provincial, district, and village levels. Selected members will be trained as community mobilization volunteers.

Focus Group Discussions: Approximately 110 focus group participants will be recruited (2 groups of 15 participants, and 10 groups of 6-8 participants). The focus groups will identify norms surrounding HIV-related stigma and attitudes towards the Women's Union and Youth Union as well as to pilot intervention components. Each focus group discussion will be tape recorded and last approximately 1 hour.

Pilot of Assessment Measures: We have developed an assessment battery from a variety of studies that will be piloted with 20 HIV-positive IDUs. We will use the iterative continuous quality improvement strategy to pilot and receive information feedback for each of the following measures: sexual HIV risk behaviors, drug related HIV risk behaviors, stigma, mental health disclosure of HIV-status, partner uptake of HIV Voluntary counseling and testing, social support, and self-reported disease status. Each participant will be asked for feedback about the procedures the assessment measures, and their reaction to having such questions asked of them. These sessions will last approximately 1 hour.

Phases II-IV: Baseline, Intervention and Evaluation

Recruitment Process:

For recruitment into our randomized trial, we will expand our recruitment strategy from our experimental intervention among HIV-negative IDUs in Thai Nguyen. (IRB protocol # H34.01.09.04A1) Our recruitment efforts will continue with the same network of formr and current users as recruiters (n = 31). Recruiters will be paid a salary, rather than an amount per participant recruited, to ensure a non-coercive environment. Training workshops will emphasize the importance of voluntary participation and supervisors will briefly interview 5% of participants at the study site to ensure that their decision to participate was completely voluntarily. Using a snowball sampling technique, recruiters will approach their current or former drug networks in a private place and distribute brochures and answer questions about our study. They will then accompany or refer subjects that are interested in participating to the study site in order to be screened. In order to maintain confidentiality of participants, HIV status as an eligibility criterion will not be revealed to outreach workers. During meetings with counselors at VCT centers in the province, the study will be described and counselors will be asked to distribute printed brochures that describe the study. Mass publicity campaigns are not planned to avoid adverse public attention toward the study which would undermine recruitment efforts and jeopardize the study.

Index Participants: Potential participants who come to the study site will be greeted by project staff and provided detailed information about the project, including study objectives, procedures, potential risks and benefits to the potential participant. Those who decide to continue with the study will be screened for eligibility and exclusion criteria. To establish eligibility, interviewes will obtain informed consent and administer a 15-minute screening questionnaire. As part of the screening interview, participants will be asked to provide a list of 10 names of their injecting networks. Additionally, the index participant must either accompany a network member to the study site or provide them with a sealed envelope containing information about the study in order to participate in the study.

Injecting Network Participants: A network inventory will be performed shortly after the screening interview of each index participant to identify the first three peers listed that meet our network participant enrollment criteria. Network members will be greeted by study staff upon arrival to the study site, and will be verified

through a database of names provided by index participants. Those who decide to continue with the study will be screened, and study staff will further describe study objective, procedures, risk and benefits to eligible participants and obtain written informed consent.
In-Depth Interviews: We will be conducting 10 in-depth interviews at each follow-up visit among Index participants who are randomized to the intervention arm and who return for their follow-up assessment. Survey interviewers will ask every 10th participant to participate in an additional interview until we have recruited 10 participants. We will also conduct brief qualitative interviews among a cohort of 3 community members in all villages every 8 months. Potential participants will be introduced to the study through the community health station staff in Thai Nguyen. These informants will be purposively selected to include men and women who are married and unmarried with different levels of education and types of employment.

Tracing Procedures: At enrollment, screeners will document the subjects' name, telephone number if available, typical sleeping location and names of contact who usually know where to find the subjects with the promise of confidentiality to not reveal the subject's involvement in the study. Subjects will be given an appointment for their next follow-up visit at each assessment visit and will be reminded during sessions of their next appointment. We have implemented a well-designed computerized follow-up program to keep track of all scheduled daily appointments and missed appointments together with contact information. If the participant is in the provincial rehabilitation center or hospital, we will conduct the follow-up visit in a private room at the center. As in our previous HIV-negative study, participants who become incarcerated will be followed up at home when released.

Study Procedures:

Pre-Screening Assessment: After informed consent is obtained, trained interviewers will administer a brief face-to-face questionnaire. The questionnaire will contain approximately 10 questions and will take approximately 5 minutes to complete. Categories of questions include demographics, injecting and sexual history and practices, injecting networks and locator information. We will test HIV among both index IDUs (HIV-positive) and their injecting partners (HIV-negative) to assess eligibility for participating in the study. We will use the WHO/CDC Protocol for VCT at project facilities. Trained counselors will perform risk assessment and explain the meaning of test results. Participants will provide blood from a finger prick and 2 rapid EIA tests will be run simultaneously. Results will be provided at the screening visit, and HIV posttest counseling will be provided according to WHO/CDC protocols. The staff physician will be consulted if other heath problems are identified and active referrals for medical care will be provided as needed. Additionally, we will offer clinical care for the diagnosis and treatment for opportunistic infections that may be related to HIV at no cost to our participants. In order for individuals to process the results of their HIV test, study enrollment will occur at a later visit; individuals who meet the study criteria will be contacted one week later and invited to return for an enrollment and baseline survey visit.

A copy of the screening questionnaire will be forwarded for IRB approval.

Baseline and Follow-up Assessments: After informed consent is obtained, a questionnaire will be administered to all participants at enrollment. Follow-up questionnaires will be conducted among all Index participants at months 3, 6, 12, 18 and 24, and among Network participants at month 24. Follow-up questionnaires will be conducted among all network participants at month 24. The questionnaire will be administered through face-to-face interviews in a private room at the project facility with trained interviewers. and will take approximately 1 hour to complete. Participants who appear influenced by drugs or otherwise not lucid will be asked to return for an interview the next day. Categories of questions include: Demographics, sexual risk behaviors, injecting behaviors, stigma, coping with HIV/AIDS, depression and other mental health issues, disclosure of HIV status, partner uptake of HIV VCT, social support, correct condom use and stage of disease. At baseline and each follow-up visit, Index participants will be asked to provide blood specimens in order to assess the stage of their HIV disease. Trained phlebotomists will take approximately 10cc of blood. Samples will be kept in mobile coolers labeled with the participant's study number and transferred to Hanoi Medical School for flow cytometry for CD4+ counts. Since HIV testing is part of our pre-screening

assessment, HIV tests will only be conducted among Network participants at month 24. As in the pre-screening assessment, participants being tested for HIV will be asked to provide blood through a finger prick.

A copy of baseline and follow-up questionnaires will be forwarded for IRB approval.

<u>In-Depth Interviews:</u> Qualitative interviews among 10 participants at each follow-up visit and among 3 community members every 8 months will be administered by trained interviewers after informed consent is obtained. All interviews will be tape recorded and will last approximately 1 hour.

Intervention Procedures:

<u>Individual level posttest counseling and support groups for HIV-positive IDUs:</u> The individual level intervention consists of a sequence of posttest counseling and support group sessions that builds on an overall staged, sequential approach to behavioral change for HIV-positive IDUs. Posttest counseling will focus on coping and disclosure. Support groups sessions will focus on HIV knowledge and skill-building while simultaneously providing social support through shared experiences of being an HIV-positive IDU. Each participant in the intervention arm will participate in two 2-hour individual posttest counseling sessions and *three* 2-hour support group meetings consisting of 6-10 participants. Support group meetings will be conducted by a team of two facilitators; counseling sessions will be conducted by one of these same facilitators.

Structural level community-based stigma reduction programs: Stigma reduction programs will begin in a village randomized to the stigma reduction arm within a week of village randomization and will continue throughout the 24 months of the trial. Programs will aim to reduce perceived community HIV stigma among HIV-positive IDUs. Our objectives are to correct misconceptions about HIV transmission; de-link PLWH from "social evils"; and promote positive messages on HIV and PLWH in the community. This will be achieved by promoting messages on video, providing opportunities for community members to interact directly with HIV infected IDUs while providing a meal service, and through opportunities for community members to engage in dialogues with community mobilization volunteers. The volunteers, acting as change agents, provide the link among the different programs. First, video presentations will feature 2 videos that challenge common misunderstandings about HIV transmission and promote positive messages on HIV-positive individuals. Each video presentation will be followed by a question-answer session on HIV/AIDS with a community mobilization volunteer. Second, meals on motorcycles (MOM) will enable community members to act on the videos' messages by facilitating interaction between PLWH and community members. While mobilizing the community to contribute food and meals, community mobilization volunteers will simultaneously engage in discussions about HIV/AIDS with community members. The two programs will be supplemented by community outreach. Three teams of community mobilization volunteers will disseminate HIV/AIDS information and answer questions through one-on-one or group discussions in the community as part of the effort to actively promote videos and MOM. All programs will be publicized through weekly public loudspeaker announcements.

<u>Methods for Dealing with Adverse Events</u>

During the informed consent process, at the end of each follow-up interview and in the beginning of each intervention session, study staff will remind participants to report any physical or social harm to the study staff immediately, so that participants may receive counseling and/or other assistance. A study psychiatrist and physician will both be available to study participants for the duration of the study; in cases where the psychiatrist or physician are not trained or equipped to treat the participant, referrals will be made. Participants also are asked whether they have suffered any social harm or adverse impact from the study as a routine part of follow-up interviews. Information on arrest, incarceration and drug overdose will be collected as part of the baseline and follow-up study questionnaires.

When an adverse event is reported to the study staff, the staff will investigate the details of the event and will reach a conclusion to whether the occurrence of the event was related to participation in the study. They will refer the participant to medical, psychological or social services as deemed appropriate, and if necessary, the investigators may take action to terminate the participant from the study. All adverse events and serious adverse events will be reported to the local Principal Investigator, Dr. Nguyen Le Minh within 24 hours orally or by email. A completed written report of the event will be sent to the Principal Investigator, Dr. Vivian Go within 72 hours, and to the National Institute on Drug Abuse Project Officer, the Data Safety Monitoring Board and to the Institutional Review Boards within one week.

Methods for Dealing with Illegal Reportable Activities

CPM has agreements with local law enforcement personnel to ensure that participants in HIV risk reduction programs are not arrested for participation in programs.

Samples Stored Beyond the End of the Study

A human biological materials repository for blood samples will be maintained at Johns Hopkins Bloomberg School of Public Health, under the direction of the PI, Vivian Go. We would like to use these specimens to test for other health-related issues that may be related to HIV, hepatitis, or other sexually transmitted diseases using unspecified testing methods. A separate informed consent for specimen storage will be administered to the participant. Participants do not have to agree to sign the additional consent form for specimen storage to participate in this research study.Blood will be stored as coded for 5 years after the study is completed. Participants who agree will also be asked if they consent to be contacted for future studies. Consent forms will be used to assure that participants agree for their sample to be stored as well as to be re-contacted in the future. There are no plans to share the samples with other investigators.

Risk/Benefits:

Phase I: Formative

Description of Risks and Measures to Minimize Risks

During the interview, some participants may feel uncomfortable when asked personal questions about their sexual and injecting behavior histories. Participants may refuse to answer any question that they are uncomfortable answering, and may stop the interview at any time.

Potential exposure to police authorities during the study may place IDUs in a situation where they receive referral to a drug treatment program. However, commitment from both national and local officials on protecting confidentiality and careful measures to assure confidentiality, we anticipate few challenges and no problems with these issues. Furthermore, we have established ties to this community, and have a working relationship with the local People's Committees and police authorities.

We will also take steps to maintain strict confidentiality of information on participants' HIV status. These steps are outlined below under the Confidentiality Assurances heading.

Description of Potential Benefits

There are no direct benefits for participation in the formative phase of the study. However, the information will help to HIV prevention interventions that may improve the health of their community.

Description of Level of Research Burden

The burden of participation should be low, as the phase primarily consists of casual discussions among community members lasting one hour and hour-long interviews with men and women.

Phases II-IV: Baseline, Intervention and Evaluation

Description of Risks and Measures to Minimize Risks

As in qualitative interviews, participants completing the baseline and follow-up interviews may have a small risk of psychological distress posed by study questions concerning HIV risk, sexual and drug using behaviors. Participants may find answering questions about these issues upsetting; these questions will be asked in as sensitive a manner as possible. If a participant experiences emotional upset during the interview, the research staff will be trained on how to handle these situations and the local principal investigator, Dr. Nguyen Le Minh will be available to speak with participants if needed.

There are minimal physical risks to giving blood, such as a slight pain when the needle is inserted and possible bruising. To reduce the possibility of pain and bruising, blood draws will be taken by trained phlebotomists, and participants will be asked to rest and take water following the blood draw. Testing for HIV may cause anxiety and stress while waiting for test results, and participants who are told they are HIV-positive may experience emotional and social problems, such as depression and stigmatization. Pre and post-testing counseling by trained counselors will be provided to minimize these potential effects. In addition, a psychiatrist will be on call should a counselor feel that a subject is in danger of harming him- or herself or others during the course of a study visit.

Potential exposure to police authorities during the study may place IDUs in a situation where they receive referral to a drug treatment program. However, commitment from both national and local officials on protecting confidentiality and careful measures to assure confidentiality, we anticipate few challenges and no problems with these issues. Furthermore, we have established ties to this community, and have a working relationship with the local People's Committees and police authorities.

Because HIV-status is an inclusion criteria for participation in our study, we will take steps to maintain the strict confidentiality on all participants' HIV status. These steps are further outlined below under the Confidentiality Assurances heading.

Description of Potential Benefits

IDUs participating in the survey and laboratory testing will receive free HIV testing and free treatment for opportunistic infections that may be related to HIV. The following treatments will be provided for opportunistic infections as appropriate: Co-trimomazol, Erythromycine, Ciprofloxacine, Flucanozole and Acyclovir. Participants may benefit from learning about HIV/STDs and ways to safely inject and have sex. IDUs in both intevention arms will also be invited to return to counselors as often as needed throughout the study period. At a community level, if the trial is successful there are potentially enormous social benefits that can be gained from the study. Specifically, at the end of our proposed study, we will have tested a multilevel intervention that is tailored to the social and risk-taking context of HIV-positive IDUs. MOH Life Gap, the primary agency for HIV programs in Vietnam, has indicated that our intervention is feasible and relevant in the Vietnamese context and could be sustained by the MOH. A demonstrated HIV risk reduction among HIV-positive IDUs may provide a model to stem the growth of the HIV-epidemic in Vietnam, which would benefit the community.

<u>Compensation</u>:

Individuals participating in any phase of the intervention will be paid the equivalent Vietnamese Dong of $6.50 US for each completed interview for the time they may have lost from work and the equivalent of $1.25 US to cover transportation to the research facility.

Disclosure/Consent Process:

Description of the Consent Process

Our research staff has prior experience obtaining informed consent from IDUs for clinical trials within this cultural context. The informed consent procedures have been designed to maximaize understanding of potential risks. All consent forms will be translated into Vietnamese and back-translated into English to ensure correct use of language.

Consent forms will be read aloud to participants by study interviewers. After reading the consent forms and prior to seeing a signature, interviewers will ask participants to summarize the study and explain the reasons why they want to participate. At this point, any misunderstandings regarding procedures, risk or benefits can be clarified. If there are cultural, literacy or political reasons why a signature is not appropriate, individuals will be allowed to mark the consent form with an "X." Informed consent will be obtained when data are collected including in-depth interviews, focus group discussion, the pre-screening assessment, and the baseline interview. At follow-up interviews participants will be reminded about the consent form that they signed initially and any misunderstandings will be clarified. Information sheets will be given to participants at each intervention session that include information about the research study.

Safety Monitoring:
Safety monitoring will be performed by an independent Data Safety Monitoring Committee comprised of researchers from Hanoi and individuals in Thai Nguyen with expertise in issues relevant to this study.

The committee will meet initially to review the protocol prior to submission to the Thai Nguyen Centre for Preventive Medicine IRB and before data collection begins, after which it will convene to review the study within one year of commencing enrollment and at least annually thereafter. Emergency meetings of the committee will be convened as needed.

At each meeting review of interim reports, the DSM Committee will review the following in a report prepared by a statistician external to the study team:

- Expected versus actual accrual rates
- Study retention rates, total and by study arm (unblinded)
- Study outcomes, as available, total and by study arm (unblinded)
- Numbers of adverse events and serious adverse events, by type, total and by study arms (unblinded)
- Any quality assurance or regulatory issues that occurred since the previous review
- Any actions or changes with respect to the study protocol.

Blind interim analyses of the data will be conducted halfway through the follow-up period, where virtually all participants will have provided 6-month behavioral interim endpoints. Interim reports will be prepared for the DSM Committee by an external statistician and will be presented to the protocol team blinded as to study arm, labeled as "A", "B", "C" "D".

Consideration will be given to stopping the study under each of the following scenarios.

- Increases in the overall number of drug use and risk behaviors for all study participants, as compared to baseline levels, that are statistically significant at the $p<0.001$ level.
- Differences in study outcomes between the control arm (receiving neither intervention) and any of the three study arms that are statistically significant at the $p<0.001$ level.

- Differences in the number of serious adverse events (calculated by collapsing all SAEs) between the control arm (receiving neither intervention) and any of the three study arms that are statistically significant at the $p < 0.001$ level. SAEs include mortality, suicide, accidents and violence.

When considering whether to stop the study, the overall risks and benefits experience as of the interim analysis date will be weighted by the protocol team. The recommendation of the DSM Committee and opinion of the study sponsor will be given heavy weight.

Adverse events on which reports are collected by the study staff include not only physical harms such as mortality, suicide, accidents (traffic and other) and violence, but also social harms, such as arrest, incarceration, harassment by the police, being expelled from school or work, and instances of discrimination. Serious adverse events are based on the US Federal Drug Administration definition and are defined as events that result in death, are life-threatening, result in hospitalization or prolongation of existing hospitalization, a persistent or significant disability, a congenital abnormality or birth defect. Other injuries or medical events may be considered to be serious adverse events, when, in the opinion of a physician, they may jeopardize the participant and may require medical or surgical intervention to prevent one of the above outcomes. In our study, drug abuse or dependency alone in the absence of other adverse impact will not be considered to be an adverse event; however, patterns of drug use behavior will be analyzed. To our knowledge, there are no applicable Vietnamese laws for reporting suicide, physical abuse, sexual abuse or intentional HIV transmission.

AEs and SAEs reported to study staff will be reported to the local PI, Dr. Nguyen Le Minh within 24 hours orally or by email, and a completed written report of the event will be sent to the PI, Dr. Vivian Go within 72 hours. A copy of the written report will also be sent to the NIDA project officer, the DSM Committee and IRBs within one week.

Reports of the DSM Committee meetings will be provided to the Thai Nguyen Centre for Preventive Medicine IRB and the Johns Hopkins Bloomberg School of Public Health IRB within 10 days.

Confidentiality Assurances:

Certificate of Confidentiality/Letters of Commitment

In Vietnam, there is no certificate of confidentiality. However, letters of commitment from authorities have been obtained that assure no breaches in confidentiality.

Data Security

All participants will receive an identification number (PID) and this number will be used for all interviews. No other information that would disclose the participant's identity will be found on any interview. Data will be kept without serostatus identification in locked cabinets at the Center for Preventive Medicine in Thai Nguyen. Only the consent form, tracker form and tracker computer will link the participant's name to the identification number. To maintain contact with participants, we will use a computerized tracking system as described in the Study Procedures section. In addition, interviewing and office staff will sign a confidentiality pledge prior to having contat with participants.

We will take steps to maintain strict confidentiality of information on participants' HIV status. To ensure that the HIV serostatus of IDUs will bot be disclosed by virtue of their participation in the study, we are not limiting enrollment to HIV-positive individuals. As part of Phase I, we will conduct focus groups among men

and women in the community, and HIV-serostatus will not be an eligibility criterion for this component of the study. Furthermore, our consent forms do not divulge that HIV status is an eligibility criterion for the intervention component. Although informed consent forms should disclose all eligibility criteria, however given the potential disclosure of sensitive information contained in the consent form (e.g., if a third party deliberately or inadvertently obtained the participant's copy of the consent form,) it would constitute a breach of confidentiality; therefore we are omitting this information from the consent form.

Our project sites are currently located in primary health centers (not HIV, VCT, infectious diseases or TB ward) to ensure that participation in our study does not disclose participants HIV and drug using status. And, our study name will be selected by local staff and will not contain any information about IDUs or HIV.

Who Will Have Access to the Data

To ensure confidentiality, electronic data will be saved on password-protected secure computers Access to security passwords will be given only to the PI and Field Director, Dr. Nguyen Le Minh at CPM. Personal identifiers will not be stored in the data set and all computers will be protected by anti-virus software. Raw data files will be destroyed in the field setting by shredding after one year from being electronically coded. Tapes of the qualitative interviews and focus group discussions will be destroyed within one year of being transcribed electronically by crushing and cutting the tapes. Blood samples will be incinerated after 5 years of storage in lab facilities.

Collaborative Agreements:

The study will be conducted in collaboration with the Thai Nguyen Center for Preventive Medicine (CPM), based on Memorandums of Understanding between JHU and the CPM. The local IRB is reviewing the research plan and consent forms. The approval letter will be forwarded to the JHU IRB when it is available.

The Thai Nguyen IRB is registered with the OHRP and has been granted Federalwide Assurance (IRB00004109; FWA00007138).

References

(1) Aceijas C, Stimson GV, Hickman M, Rhodes T. Global overview of injecting drug use and HIV infection among injecting drug users. *AIDS.* 2004;18:2295-2303.
(2) Subcommittee on HIV/AIDS Surveillance. HIV Sentinel Surveillance Report. 2003. Hanoi, Ministry of Health.
(3) Blackard J, Cohen D, Mayer K. Human immunodeficiency virus superinfections and recombination: Current state of knowledge and potential clinical consequences. *Clin Infect Dis.* 2002;34:1108-1114.
(4) Filippini P, Coppola N, Scolastico C et al. Does HIV infection favor the sexual transmission of hepatitis C? *Sex Transm Dis.* 2001;28:725-729.
(5) O'Brien T, Kedes D, Ganem D et al. Evidence of concurrent epidemics of human herpes virus 8 and human immunodeficiency virus type-1 in US homosexual men: Rates, risk factors, and relationship to Kaposi's Sarcoma. *J Infect Dis.* 1999;180:1010-1017.
(6) Wiley D, Visscher B, Grosser S et al. Evidence that anoreceptive intercourse with ejaculate exposure is associated with rapid CD4 loss. *AIDS.* 2000;14:707-715.
(7) Hong, K. T., Anh, N. T. V., and Ogden, J. Understanding HIV/AIDS related stigma and discrimination in Vietnam. 2004. Washington, DC, International Center for Research on Women.
(8) Ogden, J. and Nyblade, L. Common at its core: HIV-related stigma across contexts. 2005. Washington, D.C.: International Center for Research on Women.

(9) Khoat DV, Hong LD, An CQ, Ngu D, Reidpath DD. A situational analysis of HIV/AIDS-related discrimination in Hanoi, Vietnam. *AIDS Care.* 2005;17 Suppl 2:S181-S193.

(10) UN Country Team. Reduction of HIV/AIDS related employment discrimination in Viet Nam, Discussion Paper No. 5. 2004. Ha Noi.
