# Supplementary material for: Efficacy of a Multi-level Intervention to Reduce Injecting and Sexual Risk Behaviors among HIV-Infected People Who Inject Drugs in Vietnam: A Four-Arm Randomized Controlled Trial
Source: PLoS One. 2015 May 26;10(5):e0125909. doi: 10.1371/journal.pone.0125909 (PMC4444299; doi:10.1371/journal.pone.0125909)
Supplement: S2 Protocol — (PDF) [file pone.0125909.s004.pdf]

KẾ HOẠCH NGHIÊN CỨU

Nghiên cứu viên chính: Ts Vivian F. Go

Tên nghiên cứu: Dự phòng cho người dương tính: Thử nghiệm Can thiệp Ngẫu nhiên Đối chứng

Ở người Việt nam nghiện chích ma túy nhiễm HIV

Số tài trợ # *IRB00000594*

Câu hỏi nghiên cứu:

Nghiên cứu này là một thử nghiệm ngẫu nhiên đối chứng ở người nghiện chích ma túy (NCMT) nhiễm HIV tại Thái Nguyên, Việt Nam nhằm giảm hành vi tình dục và tiêm chích có nguy cơ cao. Chúng tôi sẽ tiến hành nghiên cứu này với sự cộng tác của Trung tâm Y tế Dự phòng tỉnh Thái Nguyên (CPM). Chúng tôi sẽ so sánh chương trình tư vấn xét nghiệm tự nguyện (TVXNTN) tiêu chuẩn với một can thiệp có thêm hợp phần hỗ trợ tâm lý xã hội cho những người NCMT nhiễm HIV. Can thiệp của chúng tôi gồm các chương trình giảm kỳ thị cộng đồng, và sẽ đánh giá cả can thiệp cấp cá nhân và cấp cộng đồng. Các câu hỏi nghiên cứu cho toàn bộ dự án là:

1. Những tác động của kỳ thị quan sát được liên quan đến HIV đối với việc tiết lộ tình trạng nhiễm HIV, hỗ trợ xã hội và đối diện với HIV ở người NCMT là gì ?
2. Vai trò của việc tiết lộ tình trạng nhiễm HIV, hỗ trợ xã hội và việc đối diện với HIV trong việc làm giảm các hành vi nguy cơ nhiễm HIV là gì ?
3. Can thiệp phối hợp cấp cộng đồng và cấp cá nhân có làm giảm hành vi tình dục và tiêm chích nguy cơ ở người NCMT nhiễm HIV hiệu quả hơn một can thiệp cấp cá nhân hoặc cấp cộng đồng đơn lẻ không và và có hiệu quả hơn TVXNTN HIV tiêu chuẩn không ?

Giả thuyết

Giả thuyết chính của chúng tôi là các hoạt động can thiệp phối hợp cấp độ cộng đồng và cá nhân có hiệu quả trong việc làm giảm hành vi tình dục và tiêm chích nguy cơ. Giả thuyết phụ là người mạng lưới của những người NCMT được chọn và các can thiệp cấp cộng đồng và cấp cá nhân sẽ giảm tỷ lệ nhiễm mới HIV sau 24 tháng.

Cơ sở lý luận:

Thông tin chung

Người NCMT nhiễm HIV chiếm một vị trí quan trọng trong nhóm nguy cơ HIV dương tính vì họ có khả năng truyền HIV qua hai đường: tình dục không bảo vệ và sử dụng ma túy không an toàn (1). Ở Việt Nam, người NCMT chiếm hơn 65% tất cả các trường hợp nhiễm HIV được báo cáo, và giám sát trọng điểm HIV cho thấy HIV tiếp tục tăng lên ở tất cả các nhóm giám sát, gồm cả người NCMT (2). Với việc tăng tỷ lệ hiện nhiễm HIV, tăng số lượng người NCMT và gái mại dâm có sử dụng ma túy, người NCMT có thể nhanh chóng ảnh hưởng đến việc lan HIV ra cộng đồng chung. Những can thiệp làm tăng kiến thức về HIV, khuyến khích tình nguyện tiết lộ tình trạng nhiễm HIV và tăng cường hỗ trợ xã hội trong nhóm người nhiễm HIV có thể giảm hành vi tiêm chích và tình dục nguy cơ, và việc giảm những hành vi nguy cơ này sẽ làm giảm việc lây truyền HIV (3-6).

Kỳ thị HIV đã đang được báo cáo rộng rãi ở Việt Nam, và là một trong những cản trở cơ bản đối với hoạt động dự phòng, chăm sóc và điều trị (7-9). Người sống chung với HIV (PLWH) ở Việt Nam thường trải qua sự cách ly xã hội nặng nề tại gia đình, cộng đồng, trường học và nơi làm việc, sự kỳ thị tại các trung tâm chăm sóc y tế, và mặc cảm (7, 10). Vì vậy, kỳ thị cản trở việc tiết lộ tình trạng nhiễm HIV cho người khác và là rào cản lớn nhất đối với hoạt động dự phòng, tiếp cận và hỗ trợ HIV ở Việt Nam.

Mục đích

Các mục đích chính của nghiên cứu này là:

1. Khảo sát định tính tác động của kỳ thị HIV quan sát được đối với việc tiết lộ tình trạng nhiễm HIV, hỗ trợ xã hội và đối diện với tình trạng nhiễm ở người NCMT, và đồng thời khảo sát vai trò của việc tiết lộ tình trạng nhiễm HIV, hỗ trợ xã hội và đối diện với tình trạng nhiễm trong việc giảm các hành vi có nguy cơ nhiễm HIV của người NCMT nhiễm HIV ở tỉnh Thái Nguyên, Việt Nam.
2. Tiến hành một thử nghiệm can thiệp ngẫu nhiên đối chứng với 4 nhóm ở người NCMT nhiễm HIV. Bốn nhóm của thử nghiệm là: 1) nhóm điều kiện có đối chứng; 2) nhóm hỗ trợ xây dựng kỹ năng và tư vấn sau xét nghiệm cấp độ cá nhân; 3) các chương trình giảm kỳ thị cấp độ cộng đồng; và 4) các hoạt động ở cả hai cấp độ cá nhân và cộng đồng.

Phương pháp nghiên cứu:

Thiết kế nghiên cứu và Cơ sở lý luận:

Giai đoạn I: Nghiên cứu định tính ban đầu:

Chúng tôi sẽ sử dụng nghiên cứu định tính nhằm tìm hiểu tác động của kỳ HIV quan sát được đối với việc tiết lộ tình trạng nhiễm HIV, hỗ trợ xã hội và đối diện với tình trạng nhiễm ở người NCMT, và đồng thời khảo sát vai trò của việc tiết lộ tình trạng nhiễm HIV, hỗ trợ xã hội và đối diện với tình trạng nhiễm trong việc giảm các hành vi có nguy cơ nhiễm HIV của người NCMT nhiễm HIV. Những số liệu này sẽ giúp thiết kế can thiệp và điều chỉnh các chỉ số được sử dụng trong các công cụ điều tra. Để đạt được mục đích này, chúng tôi sẽ tiến hành các phỏng vấn định tính với các mục đích sau:

1.) Khảo sát tác động của kỳ thị HIV đối với việc tiết lộ tình trạng nhiễm HIV, hỗ trợ xã hội và đối diện với tình trạng nhiễm và khảo sát vai trò của kỳ thị, hỗ trợ xã hội, và đối diện với tình trạng nhiễm trong việc giảm các hành vi có nguy cơ nhiễm HIV ở người NCMT nhiễm HIV.

2.) Mô tả kinh nghiệm của phụ nữ khi chồng/bạn tình của họ tiết lộ tình trạng nhiễm HIV.

3.) Mô tả thái đội và hành vi của cộng đồng đối với những cá nhân nhiễm HIV và đối với Hội Phụ nữ và Đoàn Thanh Niên.

4.) Tiến hành thử nghiệm các chỉ số đánh giá.

5.) Tiến hành thử nghiệm các hợp phần can thiệp.

Giai đoạn II-IV: Điều tra cơ bản, Can thiệp và Đánh giá

Nhằm xác định tác động phối hợp và đơn lẻ của các hoạt động can thiệp cấp độ cá nhân và cộng đồng đối với hành vi tiêm chích và tình dục, chúng tôi sẽ tiến hành một thử nghiệm can thiệp có bốn nhóm. Trong mỗi 4 huyện có số người NCMT nhiều nhất tỉnh Thái Nguyên, chúng tôi sẽ chọn ngẫu nhiên 2 xã. Trong mỗi huyện, một xã sẽ được chọn ngẫu nhiên vào chương trình giảm kỳ thị và xã còn lại sẽ đóng vai trò nhóm kiểm chứng, và trong mỗi xã (kể cả xã tham gia chương trình giảm kỳ thị lẫn xã kiểm chứng), một nửa số người NCMT chỉ số sẽ được chọn ngẫu nhiên vào nhóm can thiệp (nhóm hỗ trợ xây dựng kỹ năng và tư vấn sau xét

nghiệm tăng cường) và nửa còn lại sẽ đóng vai trò kiểm chứng. Thiết kế nghiên cứu tổng thể được thể hiện ở hình 1. Chỉ số đầu ra cơ bản trong nhóm người tham gia Chỉ số sẽ là các hành vi tình dục và tiêm chích có nguy cơ. Chỉ số đầu ra cơ bản trong nhóm người tham gia Mạng lưới sẽ là tỷ lệ nhiễm mới HIV. Các chỉ số đầu ra thứ cấp sẽ gồm kỳ thị quan sát được, sử dụng bao cao su đúng cách, tỷ lệ bạn tình đi TVXNTN, hỗ trợ xã hội quan sát được, trầm cảm, và tự báo cáo việc tiết lộ tình trạng nhiễm HIV.

Hình 1. Thiết kế nghiên cứu

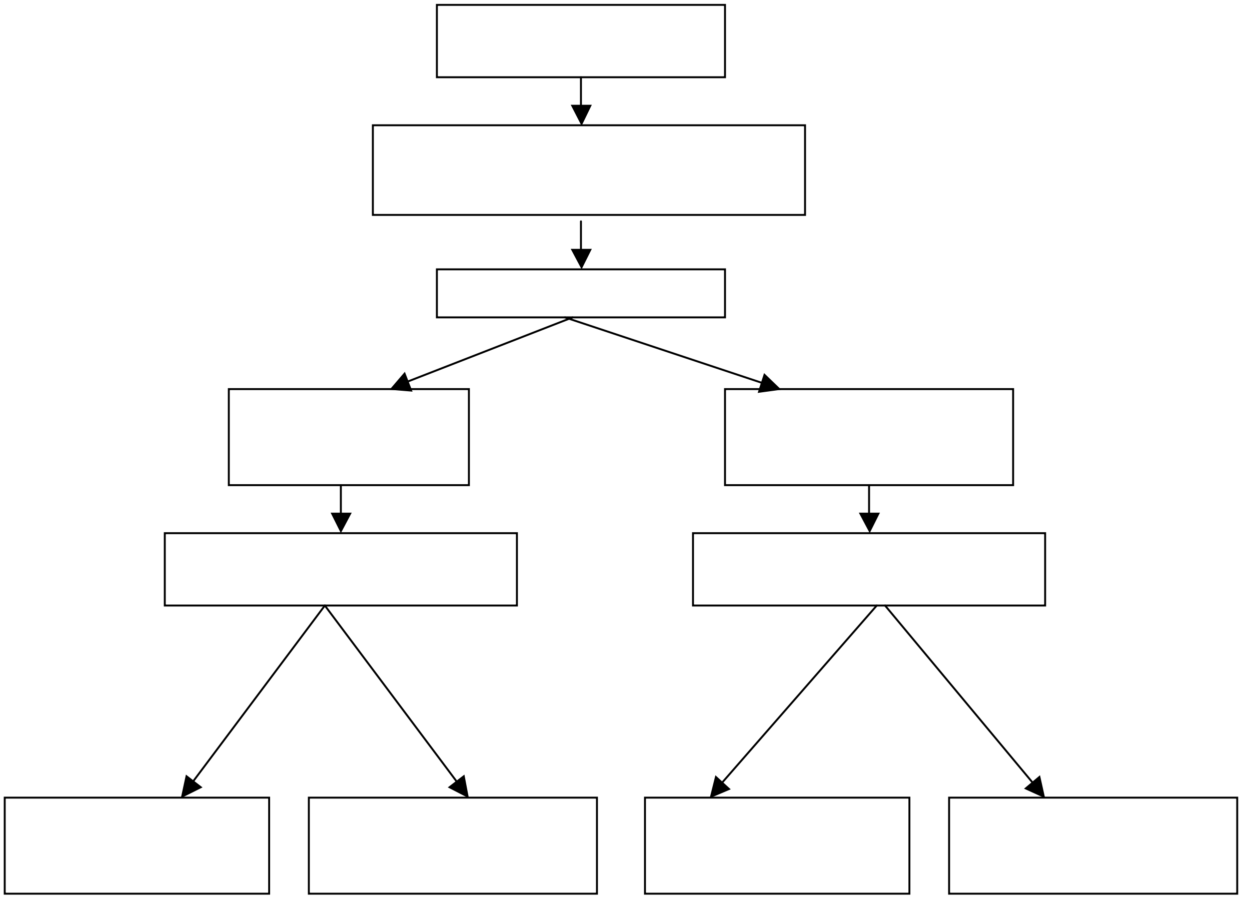

Trong số những người tham gia được chọn ngẫu nhiên vào nhóm can thiệp và những người tham gia quay lại các buổi theo dõi đánh giá định kỳ, chúng tôi sẽ tiến hành phỏng vấn định tính 10 người tham gia ở mỗi lần hẹn định kỳ. Mục đích là để tìm hiểu kinh nghiệm của người tham gia với những thay đổi hành vi tiêm chích, tình dục và kỳ thị HIV.

Nhằm đánh giá chỉ số hoạt động đầu ra của toàn bộ nghiên cứu, chúng tôi sẽ so sánh quan điểm của người tham gia về chất lượng các buổi can thiệp, tỷ lệ người NCMT tham gia vào tất cả các buổi tư vấn sau xét nghiệm và chi phí khi cung cấp dịch vụ cũng như chi phí cơ hội đối với người tham gia. Các thông tin này được thu thập qua phỏng vấn sâu với những nhóm nhỏ người tham gia, phân tích hồ sơ dự án bao gồm các mẫu đánh giá người tham gia, mẫu theo dõi tư vấn và giảng dạy cũng như các báo cáo tài chính. Đánh giá đầu ra này bao gồm đánh giá trước can thiệp đối với người tham gia và theo dõi định kỳ sau can thiệp vào tháng 3, 6, 12, 18 và 24.

Để đánh giá tỷ lệ nhiễm mới HIV ở mạng lưới bạn chích HIV- của những người NCMT nhiễm HIV, bạn chích của người NCMT nhiễm HIV đã được chọn vào thử nghiệm can thiệp sẽ được sàng lọc và xét nghiệm tình trạng HIV và được đánh giá định kỳ sau 24 tháng tính từ ngày bắt đầu tiến hành can thiệp cho người chỉ số tương ứng. Chúng tôi sẽ đề nghị người mạng lưới cho biết danh sách bạn chích và những thực hành tiêm

chích của họ với mỗi bạn chích trong vòng 2 năm qua, qua đó sẽ đánh giá khả năng phơi nhiễm HIV của người mạng lưới qua các nguồn tiêm chích khác ngoài người Chỉ số của họ.

Quần thể nghiên cứu:

Giai đoạn I: Nghiên cứu định tính

Cỡ mẫu:

Chúng tôi sẽ tuyển khoảng 170 người tham gia cho các phỏng vấn nhân chủng học, gồm người tham gia cho các cuộc phỏng vấn sâu và cho các thảo luận nhóm. Chúng tôi sẽ tiến hành 24 cuộc phỏng vấn sâu với nam NCMT nhiễm HIV và 10 phỏng vấn sâu với vợ/nữ bạn tình của những người NCMT này nhằm tìm hiểu tác động của kỳ thị HIV đối với việc tiết lộ tình trạng nhiễm, hỗ trợ xã hội và đối diện với tình trạng nhiễm trong việc giảm các hành vi nguy cơ nhiễm HIV, và tìm hiểu kinh nghiệm của người phụ nữ khi chồng/bạn tình của họ tiết lộ tình trạng nhiễm HIV. Chúng tôi cũng phỏng vấn thêm 2 nam không rõ tình trạng nhiễm HIV và NCMT để tránh khả năng cộng đồng nhận ra người NCMT nhiễm HIV mới được tham gia vào nghiên cứu này. Tương tự, chúng tôi sẽ phỏng vấn thêm 2 nữ, không rõ quan hệ mạng lưới, tình trạng HIV và tiêm chích với cùng lý do trên. Chúng tôi sẽ tiến hành 2 cuộc thảo luận nhóm nam với 8 người tham gia không rõ tình trạng HIV và tiêm chích và 2 cuộc thảo luận nhóm nữ với 8 người tham gia không rõ tình trạng HIV và tiêm chích, và một cuộc thảo luận nhóm với các lãnh đạo xã để hiểu kỳ thị HIV và đánh giá thái độ của cộng đồng đối với Hội Phụ nữ và Đoàn Thanh niên. Chúng tôi cũng tiến hành 4 thảo luận nhóm 6-8 nam và nữ trong cộng đồng để thử nghiệm các hợp phần của can thiệp cấp cộng đồng, 6 thảo luận nhóm với 6-8 nam NCMT nhiễm HIV để thử nghiệm các hợp phần can thiệp cấp cá nhân. Hơn nữa, chúng tôi sẽ thử nghiệm các chỉ số đánh giá với 20 nam NCMT nhiễm HIV.

Tính toán lực mẫu và Kế hoạch phân tích:

Vì nghiên cứu định tính tập trung vào chiều sâu hơn chiều rộng và cái nhìn sâu sắc hơn là cái nhìn chung chung nên cỡ mẫu cũng khá nhỏ so với nghiên cứu khảo sát. Cỡ mẫu phụ thuộc vào số người cần để trả lời đủ các câu hỏi nghiên cứu.

Tiêu chuẩn tham gia và loại trừ:

Phỏng vấn định tính sẽ chọn những người tham gia: 1) 18 tuổi trở lên; 2) sống tại tỉnh Thái Nguyên và 3) đồng ý tự nguyện tham gia. Chúng tôi sẽ loại trừ những người tham gia nếu họ 1) dưới 18 tuổi; 2) sống ở các xã/phường ngoài tỉnh Thái Nguyên; hoặc 3) không đồng ý tình nguyện tham gia.

Người tham gia được chọn vào phỏng vấn sâu về tác động của kỳ thị HIV cũng sẽ 1.) là nam giới; 2.) nhiễm HIV gần đây hoặc là người NCMT đã được sàng lọc trong nghiên cứu trước của chúng tôi nhưng đã không được chọn tham gia vì nhiễm HIV; 3) đã cho biết họ tình nguyện tham gia vào các nghiên cứu tương lai; 4) biết tình trạng nhiễm HIV khoảng 1 năm.

Người tham gia được chọn vào phỏng vấn sâu về kinh nghiệm của người phụ nữ khi chồng/bạn tình của họ thông báo tình trạng nhiễm HIV cũng sẽ 1) là nữ giới; và 2) là vợ/bạn tình của người tham gia NCMT nhiễm HIV.

Người tham gia được chọn vào thử nghiệm các công cụ đánh giá cũng sẽ: 1.) là người NCMT nhiễm HIV.

Giới, tuổi và nơi ở

Dựa vào số liệu của nghiên cứu HIV âm tính trước đây của chúng tôi, đại đa số người NCMT ở Thái Nguyên là nam giới (97%), quan hệ tình dục khác giới (không có trường hợp quan hệ đồng giới nào được báo cáo) và trong độ tuổi 25-29. Chúng tôi giới hạn tuổi của người tham gia từ 18 tuổi trở lên vì theo qui định của Việt Nam, người tham gia từ 18 tuổi trở lên mới có thể độc lập tự nguyện đồng ý tham gia vào nghiên cứu mà không cần có ý kiến của bố mẹ.

Các cuộc phỏng vấn sâu và thảo luận nhóm sẽ được tiến hành ở nơi riêng biệt nằm ở trung tâm tỉnh Thái Nguyên.

Giai đoạn II-IV: Điều tra cơ bản, Can thiệp và Đánh giá

Cỡ mẫu và Tính toán lực mẫu

Cho giả thuyết thứ nhất, người tham gia chỉ số NCMT được chọn vào các can thiệp cấp cá nhân và cộng đồng sẽ giảm tần suất các hành vi tiêm chích và tình dục nguy cơ, cỡ mẫu gồm 404 người NCMT nhiễm HIV có thể giúp chúng tôi phát hiện tỷ lệ giảm 40% hoặc 50% các nguy cơ tiêm chích và tình dục, với điều kiện tỷ lệ không quay lại theo dõi định kỳ là 20%. Tính toán lực mẫu dựa trên giả định phân phối điều trị theo dự định, 85% lực mẫu và alpha là .05. Với cỡ mẫu 404, chúng tôi có thể phát hiện tần suất quan hệ tình dục không bảo vệ giảm từ .40 trở lên (giả định chương trình giảm kỳ thị cũng hiểu quả như can thiệp cấp độ cá nhân; từ .50 lên nếu hiệu quả bằng 50%) đối với những phân tích về người NCMT. Nếu độ biến thiên của các tham số giữa các đường cong nguy cơ tiêm chích và tình dục tương tự như nhau, chúng tôi cũng có thể xác định tần suất sử dụng bơm kim tiêm mới tăng trên .40 cho những phân tích về người NCMT.

Cho giả thuyết thứ hai, Tỉ lệ nhiễm mới HIV ở người mạng lưới của người chỉ số được chọn và các can thiệp cấp độ cá nhân và cộng đồng sẽ giảm, chúng tôi ước tính cơ mẫu 400 (200 cho nhóm can thiệp nhận được cả 2 hoạt động can thiệp và 200 cho nhóm kiểm chứng nhận được cả 2 hoạt động kiểm chứng) ở người mạng lưới HIV âm tính sẽ giúp chúng tôi đánh giá sự khác nhau trong tỷ lệ nhiễm mới HIV ở người mạng lưới NCMT HIV âm tính, trong điều kiện tỷ lệ bỏ cuộc là 20% (160 x 2/ (1-20/100)). Với 80% lực mẫu và alpha là .05, chúng tôi có thể phát hiện tỷ lệ nhiễm mới HIV ở người mạng lưới NCMT trong 24 tháng giảm từ 20-25% trở lên.

Tiêu chuẩn tham gia và loại trừ

Để được tham gia vào nghiên cứu này, người tham gia phải có đủ các tiêu chuẩn sau: 1) chẩn đoán HIV+ được khẳng định bằng xét nghiệm trong nghiên cứu của chúng tôi; 2) lần đầu tiên biết kết quả HIV dương tính trước khi phỏng vấn dưới 30 ngày; 3) có thể và sẵn sàng giới thiệu một bạn chích tới tham gia*;* 4) là nam giới; 5) từ 18 tuổi trở lên; 6) có quan hệ tình dục trong vòng 6 tháng qua; 7) chích ma túy trong vòng 6 tháng qua; and 8) có kế hoạch sinh sống ở Thái Nguyên trong 24 tháng tới.

Tiêu chuẩn loại trừ: 1) không sẵn sàng cung cấp địa chỉ liên lạc; 2) không thể tham gia do rối loạn tâm lý, nhận thức không đầy đủ hoặc có hành vi đe dọa; hoặc 3) hiện đang tham gia một chương trình can thiệp dự phòng HIV hoặc tiêm chích khác. Khi tham gia sàng lọc, chúng tôi sẽ hỏi người tham gia xem họ đã bao giờ xét nghiệm HIV chưa, ngày và kết quả lần xét nghiệm gần nhất. Chúng tôi sẽ loại trừ phụ nữ trong nghiên cứu này vì 97% người NCMT ở Thái Nguyên là nam giới và đại đa số phụ nữ chích ma túy là gái mại dâm nên cần có các biện pháp can thiệp phù hợp với các yếu tố nguy cơ đặc trưng của họ. Chúng tôi sẽ tuyển người NCMT nhiễm HIV mới được phát hiện vì người NCMT đã biết tình trạng nhiễm HIV của mình trong một thời gian dài có thể có tâm lý và thể trạng khác với họ.

Trong quá trình tham gia phỏng vấn sàng lọc, người tham gia chỉ số sẽ được đề nghị cung cấp danh sách 10 bạn chích. Danh sách mạng lưới sẽ được thiết lập ngay sau phỏng vấn sàng lọc để xác định 3 bạn chích đáp ứng được các tiêu chuẩn tham gia mạng lưới bao gồm: 1) chích hoặc dùng chung dụng cụ tiêm chích với người chỉ số trong vòng 6 tháng qua; và 2) có liên lạc ít nhất một lần một tuần với người chỉ số. Để được tham

gia vào hợp phần can thiệp, người chỉ số sẽ được đề nghị giới thiệu được một bạn chích đủ tiêu chuẩn vào nghiên cứu. Người chỉ số có thể đi cùng người mạng lưới đến trung tâm nghiên cứu hay đưa cho họ một phong bì dán kín có các thông tin về nghiên cứu. Người mạng lưới tham gia sàng lọc sẽ được đối chiếu với cơ sở dữ liệu quản lý tên mà người chỉ số cung cấp. Những người mạng lưới quyết định tham gia nghiên cứu sẽ được sàng lọc theo các tiêu chuẩn sau: 1) HIV âm tính; 2) từ 18 tuổi trở lên; 3) chích hoặc dùng chung dụng cụ thiêm chích với người chỉ số trong vòng 6 tháng qua; và 4) có liên lạc ít nhất một lần một tuần với người chỉ số. Tiêu chuẩn loại trừ: 1) không sẵn sàng cung cấp địa chỉ liên lạc; 2) không thể tham gia do rối loạn tâm lý hoặc nhận thức không đầy đủ; hoặc 3) hiện đang tham gia một chương trình can thiệp dự phòng HIV khác. Cán bộ nghiên cứu sẽ mô tả chi tiết về mục tiêu, qui trình nghiên cứu, các nguy cơ và lợi ích của người đủ tiêu chuẩn tham gia và giải đáp các thắc mắc. Phỏng vấn viên sẽ đề nghị người mạng lưới xác nhận đồng ý tham gia, và cấp thẻ tham gia nghiên cứu.

Giới, tuổi và nơi ở

Chúng tôi sẽ loại trừ phụ nữ trong nghiên cứu này vì 97% người NCMT ở Thái Nguyên là nam giới và đại đa số phụ nữ chích ma túy là gái mại dâm nên cần có các biện pháp can thiệp phù hợp với các yếu tố nguy cơ đặc trưng của họ. Chúng tôi giới hạn tuổi của người tham gia từ 18 tuổi trở lên vì theo qui định của Việt Nam, người tham gia từ 18 tuổi trở lên mới có thể độc lập tự nguyện đồng ý tham gia vào nghiên cứu mà không cần có ý kiến của bố mẹ.

Địa điểm điều tra và can thiệp nằm ở hai khu vực riêng biệt trong thành phố Thái Nguyên nhằm đảm bảo là nhóm phỏng vấn sẽ không biết được người NCMT tham gia nhóm can thiệp nào. Địa điểm điều tra gồm hai khu nhà với 7 phòng cách biệt, cách âm nhằm tối đa số lượng người tham gia. Các phòng này được dùng để tiếp nhận người tham gia, phỏng vấn, tư vấn HIV và lấy bệnh phẩm. Chúng tôi có gắng giữ bí mật cả hai địa điểm trên (ví dụ không có biển báo, biển hiệu ở ngoài khu nhà).

Qui trình thực hiện:

Giai đoạn I: Nghiên cứu định tính

Qui trình tuyển mộ:

Chúng tôi tính số lượng người tham gia cho phỏng vấn sâu theo phương pháp chọn mẫu theo mục đích. Cụ thể, chúng tôi sẽ chọn 12 người NCMT nhiễm HIV đã tiết lộ tình trạng nhiễm của họ cho ít nhất một người và 12 người NCMT nhiễm HIV chưa tiết lộ tình trạng nhiễm HIV của mình chi bất kỳ ai. Những người này đã được sàng lọc trong nghiên cứu trước của chúng tôi nhưng không đủ tiêu chuẩn tham gia vì nhiễm HIV. Những người này đã cho biết họ muốn tham gia vào các nghiên cứu tương lai (n ~ 280). Chúng tôi chủ định chọn mẫu đối với những người NCMT đã biết tình trạng nhiễm HIV của mình khoảng 1 năm để tìm hiểu về tác động của xét nghiệm HIV lên sức khỏe tâm thần và hành vi tiêm chích ma túy và tình dục. Chúng tôi cũng chủ định chọn mẫu đối với người NCMT nhiễm HIV cho thử nghiệm các chỉ số đánh giá và các hợp phần can thiệp cấp độ cá nhân và cộng đồng. Chúng tôi cũng tuyển hai nam và hai nữ dân thường không rõ tình trạng nhiễm HIV và tiêm chích ma túy, những người này sẽ do cán bộ y tế xã phường tại Thái Nguyên giới thiệu.

Chúng tôi sẽ tuyển 10 người vợ/bạn gái của người NCMT nhiễm HIV cho phỏng vấn sâu. Những người này sẽ được chính chồng/bạn trai của họ tự nguyện giới thiệu với chúng tôi. Phỏng vấn viên sẽ hỏi người NCMT đã thông báo tình trạng nhiễm HIV cho bạn tình xem họ có sẵn sàng giới thiệu bạn tình đến phỏng vấn hay không.

Cho thảo luận nhóm về thái độ của cộng đồng, hành vi và thử nghiệm các hợp phần can thiệp cấp cộng đồng, chúng tôi sẽ tuyển nam và nữ dân thường không rõ tình trạng nhiễm HIV và tiêm chích ma túy, những người này sẽ do cán bộ y tế xã phường tại Thái Nguyên giới thiệu.

Phỏng vấn viên sẽ đọc và giả thích cho người tham gia về qui trình tự nguyện đồng ý tham gia, nếu người tham gia đồng ý, phỏng vấn viên sẽ đề nghị họ ký vào và lưu bản đồng ý tham gia tự nguyện. Chúng tôi sẽ trả lại tất cả người tham gia một bản sao của bản tự nguyện đồng ý tham gia này. Các hoạt động của dự án sẽ được tiến hành ở một nơi riêng biệt tại Thái Nguyên. Mỗi người tham gia sẽ được nhận một khoản đền bù cho khoảng thời gian họ bị mất bằng VND tương đương với $6.50 cho một cuộc phỏng vấn sâu và $6.50 cho một cuộc thảo luận nhóm.

Qui trình nghiên cứu:

<u>Phỏng vấn sâu:</u> Tổng số người tham gia gồm 24 người NCMT nhiễm HIV đã biết tình trạng của họ khoảng 1 năm, trong đó 12 người đã tiết lộ tình trạng của họ cho ít nhất một người, 12 người chưa tiết lộ tình trạng của họ cho bất kỳ ai, và 10 vợ/bạn tình của những người tham gia trên. Trong cuộc phỏng vấn sâu với người NCMT nhiễm HIV, chúng tôi sẽ tập trung tìm hiểu xem họ đã biết tình trạng nhiễm của mình trong hoàn cảnh nào, và kinh nghiệm của họ về kỳ thị, sự đối sử của xã hội cũng như các hành vi tiêm chích và tình dục trong năm qua. Mười phỏng vấn sâu với vợ/bạn tình của người NCMT nhiễm HIV sẽ tập trung vào kinh nghiệm của người phụ nữ khi chồng/bạn trai của họ tiết lộ tình trạng nhiễm. Cuộc phỏng vấn sẽ được thu băng và kéo dài khoảng 1 giờ. Chúng tôi cũng phỏng vấn 4 nam, nữ không rõ tình trạng nhiễm HIV và tiêm chính bằng những câu hỏi tương tự như cho những người tham gia thảo luận nhóm như được mô tả ở dưới đây, gồm những câu hỏi về thái độ của cộng đồng đối với người sống chung với HIV (PLWH), đối với Hội Phụ nữ và Đoàn Thanh niên. Hội Phụ nữ và Đoàn Thanh niên là những tổ chức tự nguyện phi chính phủ được thành lập vào đầu những năm 1930 ở các cấp quốc gia, tỉnh-thành phố, huyện, xã/phường. Chúng tôi sẽ tuyển và đào tạo một số người thuộc Đoàn thanh niên và Hội phụ nữ thành các tình nguyện viên vận động cộng đồng của dự án.

<u>Thảo luận nhóm:</u> Chúng tôi sẽ tuyển khoảng 110 người tham gia thảo luận nhóm (2 nhóm mỗi nhóm 15 người, và 10 nhóm mỗi nhóm 6-8 người). Thảo luận nhóm sẽ tìm hiểu các vấn đề xung quanh kỳ thị HIV, thái độ đối với Hội Phụ nữ và Đoàn Thanh niên cũng như thử nghiệm các hợp phần can thiệp. Mỗi cuộc thảo luận nhóm đều được thu băng và kéo dài khoảng 1 giờ.

<u>Thử nghiệm các Chỉ số Đánh giá</u>: Chúng tôi đã thiết kế một công cụ đánh giá dựa trên nhiều nghiên cứu khác nhau. Công cụ này sẽ được thử nghiệm với 20 người NCMT nhiễm HIV. Chúng tôi sử dụng chiến lược thử nghiệm các chỉ số và thu nhận xét một cách liên tục và theo chu kì cho từng chỉ số đánh giá sau: các hành vi tình dục nguy cơ nhiễm HIV, các hành vi tiêm chích nguy cơ nhiễm HIV, kỳ thị, sức khỏe tâm thần, tiết lộ tình trạng nhiễm HIV, tỉ lệ bạn chích đi tư vấn xét nghiệm tự nguyện, hỗ trợ xã hội và tự nguyện báo cáo tình trạng bệnh. Mỗi người tham gia sẽ được đề nghị nhận xét về quy trình, các chỉ số đánh giá và phản ứng của họ đối với mỗi câu hỏi. Mỗi phần thử nghiệm sẽ mất khoảng 1 giờ.

Giai đoạn II-IV: Nghiên cứu cơ bản, Can thiệp và Đánh giá

Qui trình tuyển chọn người tham gia:

Để tuyển chọn người tham gia cho thử nghiệm ngẫu nhiên này, chúng tôi sẽ mở rộng chiến lược so với nghiên cứu người NCMT không nhiễm HIV tại Thái Nguyên (số tài trợ # H34.01.09.04A1) Quá trình tuyển mộ sẽ được thực hiện bởi nhóm nhân viên cộng đồng đang/từng NCMT (n=31). Nhân viên cộng đồng sẽ được nhận lương thay vì nhận tiền theo số người tuyển được để đảm bảo việc tham gia của người NCMT là tự nguyện. Tập huấn cho nhân viên cộng đồng sẽ nhấn mạnh tầm quan trọng của việc tham gia tự nguyện và cán bộ giám sát sẽ nói chuyện với 5% người tham gia để đảm bảo rằng họ hoàn toàn tự nguyện quyết định tham gia nghiên cứu. Dựa vào chọn mẫu theo phương pháp "hòn tuyết lăn", nhân viên cộng đồng sẽ tiếp cận những người đã/đang NCMT mà mình biết ở một địa điểm kín đáo, phát tờ rơi và trả lời câu hỏi liên quan đến nghiên cứu. Họ sẽ giới thiệu hoặc đi cùng những người muốn tham gia tới địa điểm nghiên cứu. Nhằm đảm bảo bí mật cho người tham gia, chúng tôi sẽ không thông báo cho nhân viên cộng đồng về việc nhiễm HIV là tiêu chuẩn tham gia nghiên cứu. Chúng tôi cũng sẽ gặp các cán bộ tư vấn xét nghiệm tự nguyện ở địa phương để giới thiệu về nghiên cứu này và đề nghị họ phát các tờ rơi. Nghiên cứu này sẽ không được thông báo rộng rãi trên

các phương tiện truyền thông nhằm tránh sự chú ý không cần thiết trong cộng đồng là ảnh hưởng không tốt cho việc tuyển chọn người tham gia và cho dự án.

Người tham gia chỉ số: Người tham gia tiềm năng đến địa điểm nghiên cứu sẽ được cán bộ dự án tiếp đón và giới thiệu về nghiên cứu bao gồm mục tiêu, qui trình nghiên cứu, rủi ro và lợi ích có thể gặp. Những người quyết định tham gia sẽ được sàng lọc dựa trên tiêu chuẩn tham gia và loại trừ. Để xác định tiêu chuẩn tham gia, phỏng vấn viên sẽ xác nhận sự đồng ý tham gia của người nghiện chích ma tuý và phỏng vấn sàng lọc theo bảng hỏi (dài 15 phút). Trong quá trình phỏng vấn sàng lọc người tham gia chỉ số sẽ được đề nghị cung cấp danh sách 10 bạn chích. Sau đó, người chỉ số có thể đi cùng bạn chích đến trung tâm nghiên cứu hay đưa cho họ một phong bì dán kín có các thông tin về nghiên cứu.

Mạng lưới bạn chích: Danh sách mạng lưới sẽ được thiết lập ngay sau phỏng vấn sàng lọc người tham gia chỉ số để xác định 3 bạn chích đáp ứng được các tiêu chuẩn tham gia mạng lưới. Người mạng lưới tới địa điểm nghiên cứu tham gia sàng lọc sẽ được cán bộ dự án tiếp đón và được đối chiếu với cơ sở dữ liệu quản lý tên mà người chỉ số cung cấp. Những người mạng lưới quyết định tham gia nghiên cứu sẽ được sàng lọc, cán bộ dự án sẽ giới thiệu thêm về mục tiêu, xác nhận sự đồng ý tham gia.

Phỏng vấn sâu: Chúng tôi sẽ tiến hành 10 phỏng vấn sâu ở mỗi lần theo dõi định kỳ trong số những người tham gia được chia ngẫu nhiên vào nhóm can thiệp và quay lại theo dõi định kỳ. Phỏng vấn viên sẽ mời người tham gia thứ 10 phỏng vấn thêm cho đến khi phỏng vấn đủ 10 người. Chúng tôi cũng tiến hành phỏng vấn định tính ngắn với 1 nhóm thuần tập gồm 3 thành viên cộng đồng ở mỗi xã 8 tháng/lần, cán bộ y tế xã/phường tại Thái Nguyên sẽ giới thiệu người tham gia tiềm năng. Tất cả những người cung cấp thông tin này đều được chúng tôi chủ ý lựa chọn để đảm bảo người tham gia gồm cả nam và nữ, người đã lập gia đình và độc thân, với trình độ học vấn và nghề nghiệp khác nhau.

Qui trình theo dõi: Ở giai đoạn sàng lọc, sàng lọc viên sẽ ghi lại tên, số điện thoại (nếu có), nơi ngủ của người tham gia và tên của người liên lạc khi cần. Chúng tôi sẽ hứa sẽ không tiết lộ việc họ không tham gia vào nghiên cứu. Người tham gia sẽ được nhận phiếu hẹn cho lần theo dõi định kỳ và sẽ được nhắc hẹn trước mỗi lần hẹn đó. Chúng tôi sử dụng chương trình theo dõi được thiết kế trên máy tính để giúp theo dõi lịch hẹn theo ngày và những cuộc hẹn bị lỡ cùng với thông tin liên lạc của người lỡ hẹn. Nếu người tham gia vào trung tâm cai nghiện hoặc nằm viện, chúng tôi sẽ theo dõi định kỳ tại một phòng riêng biệt tại trung tâm. Trong nghiên cứu người NCMT HIV- trước, đối với những người tham gia đang trong trại giam, chúng tôi sẽ theo dõi định kỳ khi họ ra trại.

Qui trình nghiên cứu:

Đánh giá tiền sàng lọc: Sau khi người tham gia ký Bản đồng ý tự nguyện tham gia, phỏng vấn viên được đào tạo sẽ tiến hành phỏng vấn cá nhân theo bảng hỏi. Bảng hỏi gồm khoảng 10 câu hỏi và mất khoảng 5 phút để hoàn thành. Các câu hỏi có nội dung về nhân khẩu học, tiền sử tiêm chích và tình dục, và thực hành, mạng lưới bạn chích và thông tin liên lạc. Chúng tôi sẽ xét nghiệm HIV cho cả người tham gia chỉ số (HIV dương tính) và người mạng lưới (HIV âm tính) để xác định tiêu chuẩn tham gia nghiên cứu. Chúng tôi sẽ tiến hành tư vấn xét nghiệm tự nguyện theo hướng dẫn của WHO/CDC tại khu nghiên cứu. Khi tư vấn trước xét nghiệm, tư vấn viên đã được tập huấn sẽ đánh giá nguy cơ và giải thích ý nghĩa của kết quả xét nghiệm. Người tham gia sẽ được chích máu đầu ngón tay và đồng thời tiến hành hai test nhanh EIA. Kết quả sẽ được trả ở lần tham gia sàng lọc và tư vấn sau xét nghiệm sẽ được tiến hành theo hướng dẫn của WHO/CDC. Nếu người tham gia có các vấn đề về sức khoẻ khác chúng tôi sẽ hỏi ý kiến bác sỹ và giới thiệu họ đến các dịch vụ cần thiết. Ngoài ra chúng tôi cũng cung cấp miễn phí các chăm sóc y tế, điều trị cho các bệnh nhiễm trùng cơ hội liên quan đến HIV. Để tạo điều kiện cho người NCMT hiểu được kết quả xét nghiệm, họ sẽ được mời tham gia khi đến trung tâm nghiên cứu ở lần tiếp theo; những người đủ tiêu chuẩn nghiên cứu sẽ được liên lạc trong vòng 1 tuần để tham gia vào nghiên cứu và tham gia điều tra cơ bản

Chúng tôi sẽ gửi bản sao bộ câu hỏi sàng lọc lên Ban đạo đức nghiên cứu xin phê duyệt.

Điều tra cơ bản và đánh giá định kỳ: Sau khi người tham gia ký Bản đồng ý tự nguyện tham gia, phỏng vấn viên sẽ tiến hành phỏng vấn tất cả người tham gia được tuyển chọn theo bộ câu hỏi. Bộ câu hỏi theo dõi định kỳ sẽ được tiến hành ở tất cả người tham gia chỉ số ở tháng thứ 3, 6, 12, 18 và 24, và người mạng lưới ở tháng thứ 24. Phỏng vấn viên đã được tập huấn sẽ tiến hành phỏng vấn bảng hỏi theo cá nhân trong các phòng riêng tại khu nghiên cứu và dự kiến kéo dài khoảng 60 phút. Những người đang say thuốc, thiếu thuốc, không tỉnh táo sẽ được đề nghị quay trở lại phỏng vấn vào một ngày khác. Bảng hỏi gồm các phần: Nhân khẩu học, tình vi tình dục nguy cơ, hành vi tiêm chích, kỳ thị, đối diện với HIV/AIDS, trầm cảm và các vấn đề sức khỏe tâm thần khác, tiết lộ tình trạng nhiễm HIV, tỷ lệ bạn chích đi tư vấn xét nghiệm tự nguyện, hỗ trợ xã hội, sử dụng bao cao su đúng cách và các giai đoạn bệnh. Tại lần điều tra cơ bản và mỗi lần đánh giá định kỳ, người tham gia chỉ số sẽ được lấy máu xét nghiệm đánh giá giai đoạn bệnh HIV. Kỹ thuật viên lấy mẫu đã được đào tạo sẽ lấy khoảng 10cc máu. Mẫu bệnh phẩm sẽ được dán mã số của người tham gia, giữ trong hộp bảo quản lạnh và vận chuyển đến trường Đại học Y Hà nội đếm tế bào CD4. Vì xét nghiệm HIV là một phần trong đánh giá tiền sàng lọc của chúng tôi, người tham gia mạng lưới sẽ chỉ xét nghiệm HIV vào tháng thứ 24. Khi đánh giá tiền sàng lọc, người tham gia sẽ được lấy máu đầu ngón tay xét nghiệm HIV.

Chúng tôi sẽ gửi bản sao bộ câu hỏi nghiên cứu cơ bản và theo dõi định kỳ lên Ban đạo đức nghiên cứu xin phê duyệt.

Phỏng vấn sâu: Cứ 8 tháng một lần, phỏng vấn viên đã được huấn luyện sẽ tiến hành phỏng vấn sâu 10 người tham gia ở mỗi lần theo dõi định kỳ và 3 người dân thường sau khi đã xác nhận người tham gia tự nguyện đồng ý. Tất cả các buổi phỏng vấn đều được thu băng và kéo dài khoảng 1 giờ.

Qui trình can thiệp:

Các buổi tư vấn sau xét nghiệm và hỗ trợ nhóm đối với người NCMT nhiễm HIV: Can thiệp cấp độ cá nhân bao gồm kết quả của các buổi tư vấn sau xét nghiệm và hỗ trợ nhóm xây dựng trên bước tiếp cận sau và tổng quát đối với thay đổi hành vi. Tư vấn sau xét nghiệm sẽ tập trung vào việc đối diện và tiết lộ trình trạng nhiễm HIV. Các buổi hỗ trợ nhóm sẽ tập trung vào kiến thức về HIV và xây dựng kỹ năng trong khi đồng thời cung cấp hỗ trợ xã hội thông qua việc chia sẻ kinh nghiệm của người NCMT nhiễm HIV. Từng thành viên trong nhóm can thiệp sẽ tham dự hai buổi tư vấn sau xét nghiệm cá nhân kéo dài 2 tiếng và 3 buổi hỗ trợ nhóm trong vòng 2 tiếng bao gồm từ 6 đến 10 thành viên tham gia. Các buổi họp hỗ trợ nhóm sẽ được hai giảng viên can thiệp thực hiện; các khóa tư vấn sẽ do một các giảng viên này thực hiện.

Các chương trình giảm kỳ thị cấp độ cộng đồng: Các chương trình giảm kỳ thị cộng đồng sẽ được bắt đầu thực hiện tại một xã được chọn ngẫu nhiên cho nhóm giảm kỳ thị trong vòng một tuần chọn ngẫu nhiên làng và sau đó sẽ tiếp tục trong suốt 24 tháng của thử nghiệm. Mục đích của các chương trình này là nhằm giảm kỳ thị cộng đồng đối với HIV trong số những người NCMT HIV dương tính. Mục tiêu của chúng tôi là sửa lại những khái niệm sai về việc lây truyền HIV; tách những người sống chung với HIV (PLWH) ra khỏi khái niệm "các tệ nạn xã hội"; và tăng cường các thông điệp tích cực về HIV và PLWH trong cộng đồng. Mục đích này sẽ được thực hiện bằng cách tăng cường các thông điệp thông qua video, đem lại cơ hội cho các thành viên trong cộng đồng bằng việc trực tiếp liên lạc với những người NCMT nhiễm HIV khi cung cấp thức ăn cho họ, và thông qua các cơ hội đối với các thành viên trong cộng đồng để tham gia đối thoại với những tình nguyện viên vận động cộng đồng. Tình nguyện viên, với tư cách là tác nhân của sự thay đổi, sẽ đưa ra sự kết nối giữa các chương trình khác nhau. Thứ nhất, việc trình chiếu video sẽ được chia ra thành 2 băng video đề cập đến những cách hiểu sai thường gặp về lây truyền HIV và nâng cao các thông điệp tích cực về cá nhân những người nhiễm HIV. Sau mỗi lần chiếu sẽ có một buổi câu hỏi và trả lời về HIV/AIDS với tình nguyện viên vận động cộng đồng. Thứ hai, việc phục vụ thức ăn bằng xe máy sẽ cho phép các thành viên cộng đồng hành động trên những thông điệp của cuốn băng video bằng cách thúc đẩy mối quan hệ tương tác giữa những PLWH với các thành viên cộng đồng. Trong khi vận động cộng đồng đóng góp thức ăn và lương thực, các tình nguyện viên sẽ tiếp tục tham gia vào các thảo luận về HIV/AIDS với các thành viên cộng đồng. Hai

chương trình này sẽ được bổ sung bằng các hoạt động tiếp cận cộng đồng. Ba nhóm tình nguyện vận động cộng đồng sẽ tuyên truyền các thông tin về HIV/AIDS và trả lời câu hỏi thông qua thảo luận riêng từng người hoặc thảo luận nhóm trong cộng đồng như là một phần của nỗ lực tích cực thúc đẩy quảng bá video và phục vụ thức ăn bằng xe máy. Tất cả các chương trình đều được tuyên truyền cộng đồng bằng cách thông báo qua loa công cộng hàng tuần.

Phương pháp giải quyết sự cố

Khi thu thập Bản tự nguyện đồng ý tham gia, vào cuối mỗi buổi phỏng vấn theo dõi định kỳ và bắt đầu mỗi buổi giảng can thiệp, cán bộ nghiên cứu sẽ nhắc người tham gia báo lại bất kỳ tổn thương thể chất hoặc xã hội mà họ gặp phải để có thể được tư vấn hoặc nhận những sự trợ giúp khác. Trong suốt thời gian nghiên cứu, một bác sỹ tâm lý và một bác sỹ khám bệnh sẽ luôn luôn có mặt để hỗ trợ cho người tham gia; trường hợp người tham gia mắc một bệnh mà bác sỹ tâm lý hoặc bác sỹ khám bệnh chưa được đào tạo để điều trị thì họ sẽ được giới thiệu đến nơi khám chữa bệnh phù hợp. Trong tất cả các cuộc phỏng vấn theo dõi định kỳ, người tham gia cũng được hỏi liệu họ có phải chịu bất kỳ một sự tổn thương xã hội hoặc chịu các tác động tiêu cực từ nghiên cứu hay không. Các thông tin về tạm giam, bắt giữ, và sử dụng thuốc quá liều cũng được thu thập trong bảng hỏi điều tra cơ bản và các cuộc phỏng vấn theo dõi định kỳ.

Khi một sự cố được báo cáo cho cán bộ dự án thì cán bộ dự án sẽ tìm hiểu chi tiết về sự cố và sẽ xác định xem liệu sự cố xảy ra có phải là do sự tham gia vào nghiên cứu gây ra hay không. Cán bộ dự án sẽ giới thiệu người tham gia đến các dịch vụ y tế, tâm lý hoặc dịch vụ xã hội thích hợp, và nếu nghiên cứu viên thấy cần thiết thì có thể quyết định chấm dứt sự tham gia của người tham gia và dự án. Các sự cố và sự cố nghiêm trọng sẽ được báo cáo miệng lên Nghiên cứu viên chính tại địa phương hoặc qua thư điện tử trong vòng 24 tiếng và báo cáo bằng văn bản cho Nghiên cứu viên chính trong vòng 72 tiếng. Một bản sao của báo cáo này cũng sẽ gửi đến cho cán bộ Viện chống Ma túy Hoa Kỳ, Ban theo dõi An toàn và Dữ liệu và Ban phê duyệt Đạo đức nghiên cứu, trong vòng một tuần kể từ khi báo cáo được hoàn thành, để xem xét lại toàn bộ hoạt động của nghiên cứu.

Phương pháp giả quyết các hoạt động của pháp luật
Trung tâm Y tế Dự phòng đã thống nhất với các nhà chức trách địa phương sẽ không bắt giam những người tham gia NCMT chỉ vì họ tham gia vào chương trình nghiên cứu.

Lưu bệnh phẩm đến cuối dự án
Mẫu máu sẽ được lưu ở trường Y tế Công cộng Bloomberg, Đại học Johns Hopkins, dưới sự giám sát của nghiên cứu viên chính, Ts Vivian Go. Chúng tôi muốn dùng những bệnh phẩm này để xét nghiệm những vấn đề ảnh hưởng đến sức khỏe khác có thể liên quan đến HIV, viêm gan và các bệnh lây truyền qua đường tình dục bằng các phương pháp xét nghiệm mới. Người tham gia sẽ được giải thích về một bản tự nguyện đồng ý tham gia riêng cho việc lưu bệnh phẩm và có thể tiếp cận lại trong tương lai. Người tham gia không bị bắt buộc ký bản tự nguyện đồng ý tham gia bổ sung cho việc lưu trữ bệnh phẩm khi tham gia nghiên cứu. Các mẫu máu sẽ được ghi mã số và lưu 5 năm sau khi dự án kết thúc. Chúng tôi không có kế hoạch chia sẻ mẫu bệnh phẩm với các nghiên cứu viên khác.

Rủi ro/Lợi ích:
Giai đoạn I: Nghiên cứu định tính

Mô tả rủi ro và các phương pháp giảm thiểu rủi ro

Khi tham gia phỏng vấn, một số người tham gia có thể sẽ cảm thấy không thoải mái khi trả lời những câu hỏi nhạy cảm về tiền sử hành vi sử dụng ma túy và tình dục. Người tham gia có thể từ chối trả lời bất kỳ câu hỏi nào mà họ cảm thấy không thoải mái, và có thể rút khỏi cuộc phỏng vấn ở bất cứ thời điểm nào.

Việc để các cơ quan công an biết trong khi nghiên cứu cũng đặt những người NCMT và tình huống có thể phải đi điều trị cai nghiện ma túy. Tuy nhiên, các cơ quan chức năng địa phương cũng như quốc gia đều cam kết giữ bí mật và có những biện pháp cẩn trọng để đảm bảo bí mật, chúng tôi gần như không gặp phải vấn đề hay thách thức gì liên quan đến vấn đề này. Hơn nữa chúng tôi đã có mối quan hệ khăng khít với cộng đồng này, và đã thiết lập được mối quan hệ công việc với Ủy ban Nhân dân tỉnh cũng như cơ quan công an địa phương.

Chúng tôi cũng tiến hành các bước duy trì bảo mật thông tin về tình trạng HIV của người tham gia. Các bước này sẽ được mô tả ở Qui trình Đảm bảo Bí mật.

Mô tả lợi ích tiềm năng

Không có lợi ích trực tiếp cho việc tham gia vào giai đoạn nghiên cứu định tính. Tuy nhiên những thông tin thu được từ nghiên cứu này sẽ giúp chúng tôi xây dựng một can thiệp dự phòng HIV là cải thiện các dịch vụ y tế cho cộng đồng này.

Mô tả mức bất tiện khi tham gia

Sự bất tiện vì tham gia vào nghiên cứu này rất thấp, vì giai đoạn nghiên cứu ban đầu chỉ gồm các cuộc thảo luận thông thường với các thành viên công đồng trong khoảng 1 giờ và các buổi phỏng vấn trong khoảng 1 giờ với người tham gia nam và nữ.

Giai đoạn II-IV: Điều tra cơ bản, Can thiệp và Đánh giá

Mô tả rủi ro và các phương pháp giảm thiểu rủi ro

Giống như phỏng vấn định tính, người tham gia trong các buổi phỏng vấn điều tra cơ bản và theo dõi định kỳ có thể có nguy cơ nhỏ về việc căng thẳng tâm lý khi được hỏi các câu hỏi liên quan đến HIV, hành vi tiêm chích và tình dục. Người tham gia có thể phật ý khi trả lời các câu hỏi này; những câu hỏi này sẽ được đưa ra theo một cách thức càng tế nhị càng tốt. Trong trường hợp người tham gia cảm thấy thất vọng khi phỏng vấn, nhân viên nghiên cứu sẽ được đào tạo về cách xử lý những tình huống này và Nghiên cứu viên chính tại địa phương, Bs Nguyễn Lê Minh luôn sẵn sàng nói chuyện với người tham gia khi cần thiết.

Có những nguy cơ thể chất tối thiểu đối với việc lấy máu, chẳng hạn như hơi đau khi xuyên kim lấy màu, và có thể sưng sau lấy máu. Để giảm khả năng đau và sưng, việc lấy máu sẽ được thực hiện bởi các kỹ thuật viên lấy máu được đào tạo. Ngoài ra, người tham gia sẽ được đề nghị nghỉ ngơi và uống nước sau khi lấy máu. Xét nghiệm HIV có thể gây tâm lý lo lắng và căng thẳng khi chờ kết quả xét nghiệm. Những người tham gia được thông báo đã bị nhiễm HIV có thể sẽ trải qua những vấn đề về tâm lý và xã hội, như trầm cảm và kỳ thị. Tư vấn viên đã được huấn luyện sẽ cung cấp tư vấn trước và sau xét nghiệm nhằm giảm thiểu các ảnh hưởng tiềm năng này. Hơn nữa, chúng tôi cũng sẽ gọi cho bác sĩ tâm lý nếu tư vấn viên cảm thấy vấn người tham gia có thể tự gây nguy hiểm cho bản thân trong khi tham gia vào nghiên cứu này.

Việc để các cơ quan công an biết trong khi nghiên cứu cũng đặt những người NCMT và tình huống có thể phải đi điều trị cai nghiện ma túy. Tuy nhiên, các cơ quan chức năng địa phương cũng như quốc gia đều cam kết giữ bí mật và có những biện pháp cẩn trọng để đảm bảo bí mật, chúng tôi gần như không gặp phải vấn đề hay thách thức gì liên quan đến vấn đề này. Hơn nữa chúng tôi đã có mối quan hệ khăng khít với cộng đồng này, và đã thiết lập được mối quan hệ công việc với Ủy ban Nhân dân tỉnh cũng như cơ quan công an địa phương.

Vì tình trạng HIV là một trong các tiêu chuẩn tham gia nghiên cứu, chúng tôi cũng tiến hành các bước duy trì bảo mật thông tin về tình trạng HIV của người tham gia. Các bước này sẽ được mô tả ở Qui trình Đảm bảo Bí mật.

Mô tả các lợi ích tiềm năng

Những người NCMT tham gia vào khảo sát và xét nghiệm sẽ được xét nghiệm HIV miễn phí và điều trị miễn phí những bệnh nhiễm trùng cơ hội có thể liên quan đến HIV. Những điều trị sau đối với các nhiễm trùng cơ hội sẽ được cung cấp: Co-trimomazol, Erythromycine, Ciplrofoxacine, Fluconazole, và Acyclovir. Những người tham gia có thể thu lợi từ việc học về HIV/STD và các phương pháp tiêm chích và tình dục an toàn. Những người NCMT trong cả hai nhóm can thiệp sẽ được mời thường xuyên quay lại gặp tư vấn viên khi cần thiết trong giai đoạn nghiên cứu. Nếu thử nghiệm thành công ở cấp độ cộng đồng, có thể những lợi ích xã hội to lớn sẽ thu được từ nghiên cứu. Chúng tôi mong muốn can thiệp này sẽ có hiệu quả, bền vững, và dễ dàng tuyên truyền trong những bối cảnh tại các nước đang phát triển khác, cụ thể những nước có các nạn dịch tập trung vào người NCMT. Đặc biệt, vào cuối nghiên cứu đề xuất, chngs tôi sẻ thử nghiệm can thiệp đa cấp độ được thực hiện trong bối cảnh chấp nhận nguy cơ ở người NCMT nhiễm HIV. Life Gap Bộ Y tế, cơ quan chính thực hiện các chương trình HIV tại Việt Nam, đã cho thấy can thiệp của chúng tôi là khả thi và phù hợp với ngữ cảnh Việt Nam và có thể được Bộ Y tế Việt Nam thực hiện lâu dài. Bằng chứng giảm nguy cơ nhiễm HIV ở người NCMT có HIV có thể đưa ra một ví dụ mẫu nhằm giảm sự gia tăng dịch HIV tại Việt Nam, và điều này mang lợi cho cộng đồng.

Đền bù:

Những cá nhân tham gia vào bất kỳ giai đoạn can thiệp nào cũng sẽ nhận được một khoản đền bù bằng VND (tương đương với 6,50 đô la Mỹ) sau mỗi lần hoàn tất phỏng vấn để bù đắp cho phần thời gian họ đã mất vì không làm việc và phần chi phí cho họ đi tới nơi tiến hành nghiên cứu.

Tiết lộ/Qui trính xác nhận tự nguyện:

Mô tả qui trình xác nhận đồng ý tự nguyện tham gia.

Các nhân viên nghiên cứu của chúng tôi đã có kinh nghiệm trước về việc thu thập Bản tự nguyện đồng ý tham gia từ người NCMT cho các thử nghiệm y tế trong ngữ cảnh văn hóa này. Qui trình thu thập Bản tự nguyện đồng ý tham gia cho nghiên cứu này được thiết kế để tối đa hóa sự hiểu biết về nguy cơ tiềm năng. Tất cả các mẫu Bản tự nguyện đồng ý tham gia sẽ được dịch sang tiếng Việt và dịch ngược lại tiếng Anh nhằm đảm bảo sử dụng đúng ngôn từ.

Bản tự nguyện đồng ý tham gia sẽ được các phỏng vấn viên đọc to cho những người tham gia. Sau khi đọc Bản tự nguyện đồng ý tham gia và trước khi kiểm tra chữ ký, phỏng vấn viên sẽ đề nghị những người tham gia tóm tắt lại nghiên cứu và giải thích các lý do tại sao họ muốn tham gia. Ở điểm này, bất kỳ sự hiểu nhầm nào liên quan đến thủ tục, nguy cơ hoặc lợi ích có thể được làm rõ. Nếu có những lý do văn hóa, học vấn hoặc chính trị về việc sẽ không phù hợp khi có chữ ký, các thành viên sẽ được phép đánh dấu "X" lên Bản tự nguyện đồng ý tham gia. Sẽ có từng Bản tự nguyện đồng ý tham gia riêng cho mỗi lần thu thập dữ liệu bao gồm nghiên cứu sơ bộ, điều tra cơ bản, phỏng vấn với một số người tham gia được lựa chon vào điều tra, và các lần đến theo dõi định kỳ. Khi đến theo dõi định kỳ, người tham gia sẽ được nhắc lại về Bản tự nguyện đồng ý tham gia mà họ đã ký từ lần đến trước và giải tích bất kỳ hiểu nhầm nào. Chúng tôi sẽ phát cho người tham gia các tờ thông tin gồm các nội dung về nghiên cứu nàu vào mỗi buổi giảng can thiệp.

Kiểm soát an toàn:

Ban theo dõi số liệu và an toàn nghiên cứu (TDSLATNC) sẽ được thành lập độc lập gồm các nghiên cứu viên và cán bộ tại Hà Nội và Thái Nguyên có chuyên môn sâu liên quan đến nghiên cứu này.

Ban này sẽ họp lần đầu để xem lại hướng dẫn thực hiện dự án trước khi trình Ban phê duyệt đạo đức nghiên cứu (IRB) tại địa phương và trước khi bắt đầu thu thập số liệu, và sẽ họp lại để đánh giá nghiên cứu trong vòng một năm kể từ khi bắt đầu tuyển người tham gia và sau đó, họp ít nhất mỗi năm một lần. Nếu cần thiết, Ban này sẽ triệu tập các buổi họp khẩn cấp.

Tại mỗi buổi họp lại, Ban TDSLATNC sẽ xem xét một báo cáo tạm thời do một nhà thống kê ngoài nhóm nghiên cứu chuẩn bị, trong đó bao gồm những nội dung sau:

- Tỷ lệ thực tế và tỷ lệ kỳ vọng;
- Tỷ lệ người tham gia quay lại, tỷ lệ tổng số và tỷ lệ theo từng nhóm (không mù),
- Kết quả nghiên cứu, tổng số và theo từng nhóm (không mù)
- Số lượng các sự cố và những sự cố nghiêm trọng, theo từng loại, tổng số và theo từng nhóm (không mù)
- Các vấn đề liên quan đến việc đảm bảo chất lượng hoặc quy định đã xảy ra trong năm
- Các hành động hoặc thay đổi liên quan đến sự tuân thủ đề cương nghiên cứu

Các phân tích mù, tạm thời sẽ được thực hiện từ khi giai đoạn theo dõi định kỳ tiến hành được một nửa (và khi phần lớn người tham gia đã cung cấp các thông tin về hành vi của trong vòng 6 tháng qua). Những báo cáo tạm thời này sẽ do một cán bộ thống kê bên ngoài (như đã đề cập ở trên) chuẩn bị cho Ban TDSLATNC; và sẽ không đề tên nhóm nghiên cứu khi trình bầy cho nhóm xây dựng nghiên cứu (các nhóm nghiên cứu sẽ được đặt tên đơn giản hóa như A, B, C và D).

Cân nhắc việc dừng nghiên cứu sẽ dựa trên các yếu tố sau:

- Sự gia tăng về hành vi nguy cơ và NCMT trong số những người tham gia so với số liệu điều tra ban đầu có ý nghĩa thống kê với $p<.001$;
- Sự khác biệt trong kết quả nghiên cứu giữa nhóm kiểm chứng (không có các hoạt động can thiệp) và một trong 3 nhóm còn lại có ý nghĩa thống kê với $p<.001$;
- Sự khác biệt về số lượng các sự cố nghiêm trọng (được tính bằng tổng tất cả các sự cố) giữa nhóm kiểm chứng (không có hoạt động can thiệp) và một trong 3 nhóm còn lại có ý nghĩa thống kê với $p<.001$. Các sự cố nghiêm trọng bao gồm tử vong, tự sát, tai nạn và bạo lực.

Khi cân nhắc việc dừng nghiên cứu, nhóm nghiên cứu sẽ cân nhắc tất cả các yếu tố có hại và có lợi cho người tham gia được thể hiện trong bản phân tích tạm thời. Những đề xuất của Ban TDSLATNC và ý kiến của nhà tài trợ sẽ được cân nhắc nhiều hơn

Các sự cố được cán bộ dự án tổng hợp trong báo cáo không chỉ là những tổn thương về mặt thể chất như tử vong, tự tử, tai nạn (tại nạn giao thông hoặc tai nạn khác) và bạo lực mà còn có cả những tổn thương về mặt xã hội như bị cảnh sát điều tra, bắt giữ hoặc giam giữ, bị đuổi học hoặc đuổi việc và bị phân biệt đối sử. Các sự cố nghiêm trọng được định nghĩa theo Cơ quan quản lý thực phẩm, dược phẩm Mỹ (FDA) là các nguyên nhân dẫn tới tử vong, các bệnh hiểm nghèo cần phải nằm viện hoặc nằm viện lâu ngày, hoặc là những nguyên nhân gây thương tật vĩnh viễn, khuyết tật bẩm sinh. Vết thương hoặc các trường hợp y học khác có thể được coi như là những sự cố nghiêm trọng nếu theo ý kiến của bác sỹ, chúng có thể gây hại cho người tham gia và có thể cần đến can thiệp bằng thuốc hoặc phẫu thuật để phòng một trong những sự cố nêu trên. (Với các mục đích của nghiên cứu này, chỉ nghiện ma túy hoặc phụ thuộc thuốc mà không có biểu hiện phụ khác sẽ không được coi là một sự cố, tuy nhiên các loại hành vi nghiện chích sẽ được phân tích như đã được mô tả ở trên). Theo như chúng tôi được biết, tại Việt Nam chưa có luật nào áp dụng cho việc báo cáo tự tử, bạo hành, lạm dụng tình dục hoặc cố tình làm lây truyền HIV.

Các sự cố và sự cố nghiêm trọng sẽ được báo cáo miệng lên Nghiên cứu viên chính tại địa phương hoặc qua thư điện tử trong vòng 24 tiếng và báo cáo bằng văn bản cho Nghiên cứu viên chính trong vòng 72 tiếng. Một bản sao của báo cáo này cũng sẽ gửi đến cho cán bộ Viện chống Ma túy Hoa Kỳ, Ban Theo dõi Số liệu và An toàn Nghiên cứu và Ban phê duyệt Đạo đức nghiên cứu, trong vòng một tuần kể từ khi báo cáo được hoàn thành, để xem xét lại toàn bộ hoạt động của nghiên cứu.

Báo cáo của buổi họp Ban TDSLATNC sẽ được nộp cho Ban Phê duyệt Đạo đức Nghiên cứu tại Trung Tâm Y tế Dự phòng Thái Nguyên và trường Y tế Công cộng Bloomberg, đại học Johns Hopkins trong vòng 10 ngày.

Đảm bảo tính bảo mật:

Chứng nhận bảo mật/Thư Cam kết

Việt nam không có Chứng nhận bảo mật. Tuy nhiên chúng tôi sẽ xin thư cam kết của chính quyền đảm bảo không vi phạm tính bảo mật.

An toàn số liệu

Tất cả người tham gia sẽ được nhận một số mã số tham gia (PID), mã số này sẽ được sử dụng trong toàn bộ các cuộc phỏng vấn. Trong tất cả các cuộc phỏng vấn sẽ không được để lộ bất kỳ thông tin nhận dạng nào khác của người tham gia. Dữ liệu sẽ được lưu giữ không ghi số nhận dạng, được để trong tủ file có khóa tại Trung tâm Y tế Dự phòng Thái Nguyên. Chỉ có Bản tự nguyện đồng ý tham gia, mẫu theo dõi và máy tính lưu giữ mới nối tên của người tham gia với số nhận dạng. Nhằm duy trì liên lạc với những người tham gia, chúng tôi sẽ sử dụng một hệ thống theo dõi trên máy tính, được mô tả ở trên. Ngoài ra, nhân viên phỏng vấn và nhân viên văn phòng sẽ phải ký "cam kết bảo mật " trước khi liên lạc với những người tham gia.

Chúng tôi sẽ tiến hành các bước nhằm duy trì bảo mật thông tin về tình trạng HIV của người tham gia. Nhằm đảm bảo tình trạng HIV của những người NCMT sẽ không bị tiết lộ do tham gia vào nghiên cứu, nghiên cứu của chúng tôi sẽ không chỉ giới hạn ở việc tuyển các cá nhân nhiễm HIV. Cụ thể, một phần trong nghiên cứu định tính, chúng tôi sẽ tiến hành các thảo luận nhóm nam và nữ trong cộng đồng; tình trạng HIV sẽ không phải là tiêu chuẩn lựa chọn đối với hợp phần nghiên cứu này. Hơn nữa, chúng tôi sẽ sử dụng Bản tự nguyện đồng ý tham gia không tiết lộ rằng tình trạng HIV là tiêu chuẩn lựa chọn cho hợp phần can thiệp của nghiên cứu. Mặc dù việc có thể tiết lộ thông tin nhạy cảm được thể hiện ngay trong Bản tự nguyện đồng ý tham gia (chẳng hạn như, nếu một bên thứ ba cố tình hoặc vô tình có được bản sao đơn chấp thuận của người tham gia), thì việc đó cũng cấu thành nên vi phạm bảo mật; do đó chúng tôi sẽ tránh đề cập đến những thông tin này trong Bản tự nguyện đồng ý tham gia.

Khu nghiên cứu của chúng tôi nằm tại trung tâm y tế chính (nhưng không phải trung tâm HIV, tư vấn xét nghiệm tự nguyện, các bệnh truyền nhiễm hoặc Lao) để đảm bảo việc tham gia nghiên cứu không tiết lộ tình trạng nghiện chích và HIV của người tham gia.

Người có thẩm quyền tiếp cận số liệu dự án

Để đảm bảo tính bí mật, dữ liệu điện tử sẽ được lưu trữ trong máy tính và có khẩu lệnh bảo vệ. Chỉ nghiên cứu viên chính và Bs Nguyễn Lê Minh, giám đốc Trung tâm Y tế dự phòng mới biết khẩu lệnh này. Mã số cá nhân sẽ không được lưu trong bộ dữ liệu và tất cả các máy tính đều có phần mền diệt virus. Chúng tôi sẽ hủy bằng cách cắt nhỏ giấy tờ số liệu thô tại địa bàn nghiên cứu khi chúng đã được chuyển thành dạng điện tử sau 1 năm. Băng thu các cuộc phỏng vấn chuyên sâu và thảo luận nhóm sau khi đã được gỡ băng thành file điện tử sẽ được hủy bằng cách nghiến và cắt. Mẫu máu sẽ được lưu lại phòng xét nghiệm sau 5 năm.

Thỏa thuận hợp tác:

Chúng tôi thực hiện nghiên cứu này với sự hợp tác của Trung tâm Y tế Dự phòng Thái Nguyên (CPM), dựa trên Bản ghi nhớ giữa JHU và CPM. Ban phê duyệt đạo đức nghiên cứu đại phương đang xem xét bản Kế hoạch nghiên cứu và các Bản tự nguyện đồng ý tham gia. Chúng tôi sẽ chuyển thư phê duyệt đến Ban phê duyệt đạo đức nghiên cứu JHU ngay khi có thể.

Ban phê duyệt đạo đức nghiên cứu Thái Nguyên đã được đăng ký với Ủy ban bảo vệ nghiên cứu con người (OHRP) và có Bảo hiểm toàn liên bang Mỹ (số IRB00004109; FWA00007138).

Tài liệu tham khảo

1) Aceijas C, Stimson GV, Hickman M, Rhodes T. Global overview of injecting drug use and HIV infection among injecting drug users. *AIDS.* 2004;18:2295-2303.
2) Subcommittee on HIV/AIDS Surveillance. HIV Sentinel Surveillance Report. 2003. Hanoi, Ministry of Health.
3) Blackard J, Cohen D, Mayer K. Human immunodeficiency virus superinfections and recombination: Current state of knowledge and potential clinical consequences. *Clin Infect Dis.* 2002;34:1108-1114.
4) Filippini P, Coppola N, Scolastico C et al. Does HIV infection favor the sexual transmission of hepatitis C? *Sex Transm Dis.* 2001;28:725-729.
5) O'Brien T, Kedes D, Ganem D et al. Evidence of concurrent epidemics of human herpes virus 8 and human immunodeficiency virus type-1 in US homosexual men: Rates, risk factors, and relationship to Kaposi's Sarcoma. *J Infect Dis.* 1999;180:1010-1017.
6) Wiley D, Visscher B, Grosser S et al. Evidence that anoreceptive intercourse with ejaculate exposure is associated with rapid CD4 loss. *AIDS.* 2000;14:707-715.
7) Hong, K. T., Anh, N. T. V., and Ogden, J. Understanding HIV/AIDS related stigma and discrimination in Vietnam. 2004. Washington, DC, International Center for Research on Women.
8) Ogden, J. and Nyblade, L. Common at its core: HIV-related stigma across contexts. 2005. Washington, D.C.: International Center for Research on Women.
9) Khoat DV, Hong LD, An CQ, Ngu D, Reidpath DD. A situational analysis of HIV/AIDS-related discrimination in Hanoi, Vietnam. *AIDS Care.* 2005;17 Suppl 2:S181-S193.
10) UN Country Team. Reduction of HIV/AIDS related employment discrimination in Viet Nam, Discussion Paper No. 5. 2004. Ha Noi.
