# Supplementary material for: Efficacy of a Multi-level Intervention to Reduce Injecting and Sexual Risk Behaviors among HIV-Infected People Who Inject Drugs in Vietnam: A Four-Arm Randomized Controlled Trial
Source: PLoS One. 2015 May 26;10(5):e0125909. doi: 10.1371/journal.pone.0125909 (PMC4444299; doi:10.1371/journal.pone.0125909)
Supplement: S1 Table — (DOCX) [file pone.0125909.s005.docx]

| **S1 Table. Percent and standard error (se) of index participants who had shared any**  **injecting needles, syringes or equipment in the past 3 months,**  **stratified by knowledge of HIV status at baseline** | | | | | | |
| --- | --- | --- | --- | --- | --- | --- |
| Visit No. | Baseline | 6-Month | 12-Month | 18-Month | 24-Month |  |
| 1. **for participants who knew their status at baseline** | |  |  |  |  |  |
| **Control** |  |  |  |  |  |  |
| *(No. observed)* | *(32)* | *(29)* | *(27)* | *(25)* | *(24)* |  |
| Observed data: %(se) | 78 (7) | 21 (8) | 22 (8) | 0 (0) | 0 (0) |  |
| Calibrated: %(se) | 84 (13) | 32 (20) | 22 (8) | 0 (0) | 0 (0) |  |
| **Community Intervention Only** |  |  |  |  |  |  |
| *(No. observed)* | *(32)* | *(29)* | *(25)* | *(23)* | *(23)* |  |
| Observed data: %(se) | 72 (8) | 10 (6) | 12 (7) | 0 (0) | 0 (0) |  |
| Calibrated: %(se) | 72 (8) | 10 (6) | 12 (7) | 0 (0) | 0 (0) |  |
| **Individual Intervention Only** |  |  |  |  |  |  |
| *(No. observed)* | *(28)* | *(23)* | *(20)* | *(19)* | *(19)* |  |
| Observed data: %(se) | 46 (10) | 17 (8) | 10 (7) | 0 (0) | 5 (5) |  |
| Calibrated: %(se) | 72(14) | 18 (18) | 1(1) | 0 (0) | 24 (24) |  |
| **Combined Intervention** |  |  |  |  |  |  |
| *(No. observed)* | *(25)* | *(21)* | *(20)* | *(18)* | *(19)* |  |
| Observed data: %(se) | 60 (10) | 5 (5) | 5 (5) | 17 (9) | 5 (5) |  |
| Calibrated: %(se) | 77 (16) | 0 (0) | 0 (0) | 14 (6) | 1 (1) |  |

| 1. **for participants who did not know their status at baseline** | |  |  |  |  |  |
| --- | --- | --- | --- | --- | --- | --- |
| **Control** |  |  |  |  |  |  |
| *(No. observed)* | *(57)* | *(46)* | *(41)* | *(35)* | *(40)* |  |
| Observed data: %(se) | 74 (6) | 17 (6) | 7 (4) | 3 (3) | 2 (2) |  |
| Calibrated: %(se) | 69 (8) | 15 (5) | 5 (3) | 2 (2) | 2 (2) |  |
| **Community Intervention Only** |  |  |  |  |  |  |
| *(No. observed)* | *(107)* | *(84)* | *(66)* | *(57)* | *(55)* |  |
| Observed data: %(se) | 78 (4) | 18 (4) | 12 (4) | 9 (4) | 7 (4) |  |
| Calibrated: %(se) | 78 (4) | 18 (4) | 12 (4) | 7 (3) | 7 (4) |  |
| **Individual Intervention Only** |  |  |  |  |  |  |
| *(No. observed)* | *(67)* | *(53)* | *(42)* | *(37)* | *(39)* |  |
| Observed data: %(se) | 67 (6) | 21 (6) | 14 (5) | 11 (5) | 3 (3) |  |
| Calibrated: %(se) | 70(8) | 26 (10) | 19 (12) | 13 (10) | 10 (10) |  |
| **Combined Intervention** |  |  |  |  |  |  |
| *(No. observed)* | *(107)* | *(92)* | *(86)* | *(78)* | *(78)* |  |
| Observed data: %(se) | 80 (4) | 14 (4) | 8 (3) | 9 (3) | 1 (1) |  |
| Calibrated: %(se) | 78 (6) | 13 (4) | 8 (4) | 11 (5) | 3 (3) |  |
