# Supplementary material for: Efficacy of a Multi-level Intervention to Reduce Injecting and Sexual Risk Behaviors among HIV-Infected People Who Inject Drugs in Vietnam: A Four-Arm Randomized Controlled Trial
Source: PLoS One. 2015 May 26;10(5):e0125909. doi: 10.1371/journal.pone.0125909 (PMC4444299; doi:10.1371/journal.pone.0125909)
Supplement: S2 Table — (DOCX) [file pone.0125909.s006.docx]

| **S2 Table. Percent and standard error (se) of index participants who had unprotected sex in the past 3 months,**  **stratified by knowledge of HIV status at baseline** | | | | | | |
| --- | --- | --- | --- | --- | --- | --- |
| Visit No. | Baseline | 6-Month | 12-Month | 18-Month | 24-Month |  |
| 1. **for participants who knew their status at baseline** | |  |  |  |  |  |
| **Control** |  |  |  |  |  |  |
| *(No. observed)* | *(32)* | *(29)* | *(27)* | *(25)* | *(24)* |  |
| Observed data: %(se) | 9 (5) | 3 (3) | 0 (0) | 4 (4) | 8 (6) |  |
| Calibrated: %(se) | 26 (17) | 0 (0) | 0 (0) | 4 (4) | 8 (6) |  |
| **Community Intervention Only** |  |  |  |  |  |  |
| *(No. observed)* | *(32)* | *(29)* | *(25)* | *(23)* | *(23)* |  |
| Observed data: %(se) | 6 (4) | 0 (0) | 12 (7) | 4 (4) | 0 (0) |  |
| Calibrated: %(se) | 6 (4) | 0 (0) | 12 (7) | 4 (4) | 0 (0) |  |
| **Individual Intervention Only** |  |  |  |  |  |  |
| *(No. observed)* | *(28)* | *(23)* | *(20)* | *(19)* | *(19)* |  |
| Observed data: %(se) | 18 (7) | 0 (0) | 0 (0) | 5 (5) | 0 (0) |  |
| Calibrated: %(se) | 8 (8) | 0 (0) | 0 (0) | 5 (5) | 0 (0) |  |
| **Combined Intervention** |  |  |  |  |  |  |
| *(No. observed)* | *(25)* | *(21)* | *(20)* | *(18)* | *(19)* |  |
| Observed data: %(se) | 16 (7) | 5 (5) | 5 (5) | 0 (0) | 5 (5) |  |
| Calibrated: %(se) | 1 (1) | 3 (3) | 3 (3) | 0 (0) | 0 (0) |  |
| 1. **for participants who did not know their status at baseline** | |  |  |  |  |  |
| **Control** |  |  |  |  |  |  |
| *(No. observed)* | *(57)* | *(46)* | *(41)* | *(35)* | *(40)* |  |
| Observed data: %(se) | 28 (6) | 4 (3) | 5 (3) | 0 (0) | 5 (3) |  |
| Calibrated: %(se) | 32 (8) | 7 (5) | 10 (8) | 0 (0) | 12 (8) |  |
| **Community Intervention Only** |  |  |  |  |  |  |
| *(No. observed)* | *(107)* | *(84)* | *(66)* | *(57)* | *(55)* |  |
| Observed data: %(se) | 27 (4) | 4 (2) | 3 (2) | 2 (2) | 2 (2) |  |
| Calibrated: %(se) | 27 (4) | 4 (2) | 2 (2) | 3 (3) | 2 (2) |  |
| **Individual Intervention Only** |  |  |  |  |  |  |
| *(No. observed)* | *(67)* | *(53)* | *(42)* | *(37)* | *(39)* |  |
| Observed data: %(se) | 25 (5) | 6 (3) | 2 (2) | 3 (3) | 0 (0) |  |
| Calibrated: %(se) | 29 (8) | 1 (1) | 0 (0) | 10 (10) | 0 (0) |  |
| **Combined Intervention** |  |  |  |  |  |  |
| *(No. observed)* | *(107)* | *(92)* | *(86)* | *(78)* | *(78)* |  |
| Observed data: %(se) | 30 (4) | 9 (3) | 3 (2) | 5 (3) | 3 (2) |  |
| Calibrated: %(se) | 26 (5) | 7 (3) | 1 (1) | 8 (5) | 4 (3) |  |
